# Synthesis Strategies and Multi-field Applications of Nanoscale High-Entropy Alloys

**DOI:** 10.1007/s40820-025-01779-0

**Published:** 2025-05-30

**Authors:** Bin Zhang, Qingxue Mu, Ye Pei, Siyu Hu, Shuo Liu, Taolei Sun, Guanbin Gao

**Affiliations:** 1 State Key Laboratory of Advanced Technology for Materials Synthesis and Processing, Wuhan University of Technology, 122 Luoshi Road, Wuhan, 430070 People’s Republic of China; 2 Hubei Key Laboratory of Nanomedicine for Neurodegenerative Diseases, School of Chemistry, Chemical Engineering and Life Science, Wuhan University of Technology, 122 Luoshi Road, Wuhan, 430070 People’s Republic of China

**Keywords:** High-entropy alloys, Nanoscale, Synthesis, Application

## Abstract

Alloying strategies have proven effective in enhancing the properties of metallic materials. However, conventional alloying strategies face significant limitations in preparing nanoscale multi-alloys and continuous optimizing surface-active sites. High-entropy alloys (HEAs) display a broader spectrum of unique properties due to their complex electron distribution and atomic-level heterogeneity arising from the stochastic mixing of multiple elements, which provides a diverse array of binding sites and almost continuous distribution of binding energies. This review aims to summarize recent research advancements in synthesis strategies and multi-field applications of nanoscale HEAs. It emphasizes several commonly employed synthesis strategies and significant challenges in synthesizing nanoscale HEAs. Finally, we present a comprehensive analysis of the advantages of HEAs for multi-field applications, emphasizing significant application trends related to nanosizing and multidimensionalization to develop more efficient nanoscale HEAs.

## Highlights

- The comprehensive overview of the synthesis of nanoscale high-entropy alloys and the advantages over conventional alloys.
- The comprehensive overview of the multi-field applications of nanoscale high-entropy alloys and recent cutting-edge research advances.
- The comprehensively analyses of the challenges and opportunities in high-entropy alloy development and emphasizes the key application trends in nanosizing and multidimensionalization.

## Graphical abstract

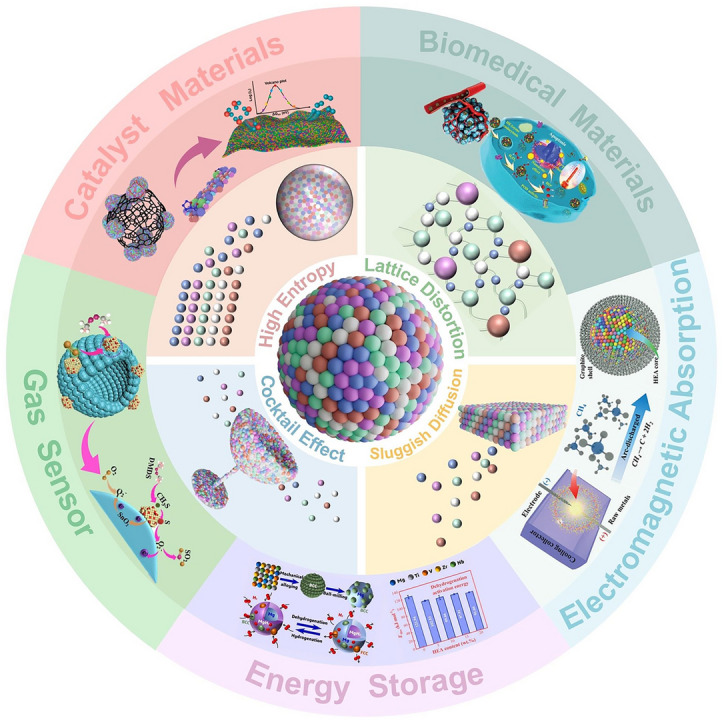

## Introduction

High-entropy alloys (HEAs) represent a novel concept that has developed rapidly within the last two decades and has expanded into various other compound classes, garnering increasing attention for their unique physicochemical properties [1]. Traditionally, the alloying strategy involved combining a small number of minor elements with a predominant major element, where one or two elements formed the bulk of the material, and the others were added to introduce specific properties [2–5]. However, the HEAs proposed by Yeh et al. and Cantor et al. in 2004 challenge this convention by lacking a dominant element. Instead, they primarily form single-phase solid solutions containing five or more elements, each with concentrations between 5 and 35% [6, 7]. Unlike traditional alloys, which tend to exhibit the properties of dominant element, HEAs display a high degree of compositional flexibility and structural diversity due to the absence of a principal element. This results in a broader range of distinctive properties [8, 9]. This innovative alloy design concept surpasses the limitations of conventional material science, offering immense potential for the discovery and development of new material properties.

The physicochemical properties of HEAs are closely related to elemental composition, surface morphology, internal structure, and interfaces. The selection of constituent elements and compositional ratios can significantly affect the distribution pattern of adsorption energy, thereby modulating the catalytic activity of the material in turn [10]. Similarly, surface morphology plays a critical role in determining the material’s properties and potential applications [11, 12]. Conventional synthesis methods for bulk HEAs predominantly employ a top-down approach, including techniques such as laser ablation [13], arc melting [14], mechanical alloying [15], and high-energy ball milling [16]. However, HEAs produced by these methods are generally bulk-sized and suffer from a considerable reduction in surface-active sites. As a result, most HEAs synthesized via these techniques are utilized primarily for their physical properties, such as exceptional thermal stability, corrosion resistance, and mechanical strength, which make them ideal candidates for applications requiring high wear resistance and low friction [17, 18]. In contrast, nanoscale HEAs can be synthesized using bottom-up strategies such as hydrothermal or one-pot methods. Advances in synthesis technologies have enabled the reduction in HEAs size, exposing a greater number of surface atoms and making them highly promising for an expanding range of applications, including catalysis [19], biomedicine [20], and energy storage [21]. Moreover, the application of specific reaction conditions during HEAs preparation facilitates the equilibrium of reduction potentials among different metals, thereby improving control over the homogeneity of HEAs nanocrystals and the degree of internal atomic ordering [22, 23]. These structures span various configurations, including solid solutions [24], intermetallic compounds [25], amorphous phases [26], and quasicrystals [27]. Furthermore, optimizing HEAs surfaces through surface ligand modification [28], surface reconstruction [29], or phase-interface design [30], which selectively exposes relevant active sites, provides an effective strategy for enhancing their physicochemical properties.

The unique elemental composition of HEAs results in distinctive physicochemical properties, and their multicomponent properties enable the manipulation of surface states, thereby creating new active sites that enhance the adsorption energy of reactants and intermediates. This characteristic makes HEAs highly promising for applications in electrocatalysis [31]. The high mechanical strength and exceptional fracture toughness of HEAs also render them suitable for use in extreme environments, such as those encountered in the aerospace and machinery manufacturing industries. The development of high-temperature-resistant engine materials and high-hardness milling cutter materials requires the stabilization of specialized materials under extreme conditions, including high temperature and pressure [32]. Moreover, in the field of energy storage, the multicomponent and high-entropy characteristics of HEAs offer the potential to significantly improve the cycling performance of batteries compared to traditional dopant materials [33, 34].

**Fig. 1:**
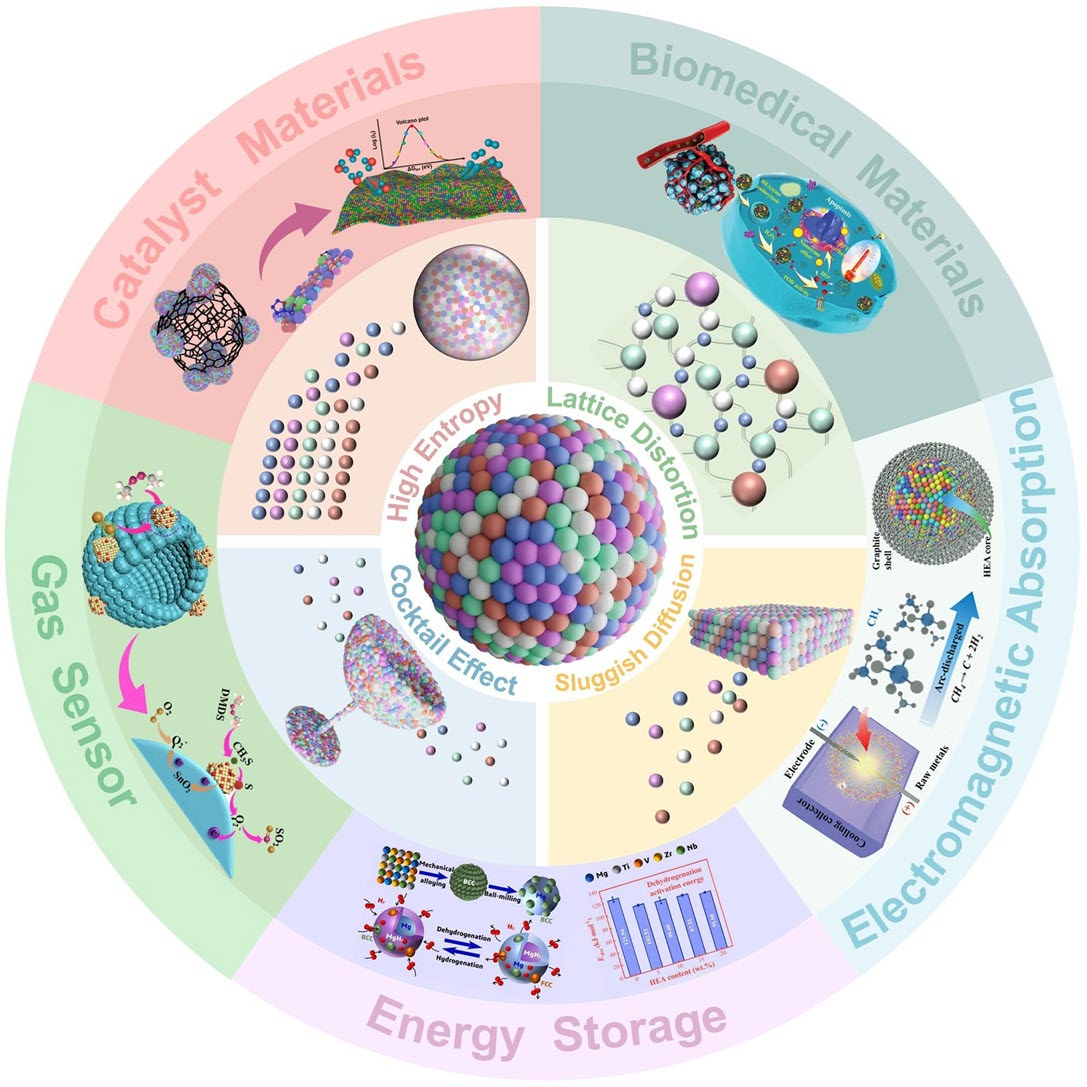
Schematic illustration of the properties of HEAs and their multi-field applications. Reproduced with permission [20, 35]. Copyright 2023, 2021, Wiley. Reproduced with permission [36–38]. Copyright 2024, 2021, 2023, Elsevier. Reproduced with permission [39]. Copyright 2022, American Chemical Society

In light of the rapid development of HEAs in recent years, this paper provides an overview of recent advances in the controlled synthesis of nanoscale HEAs and multi-fields applications (Fig. 1). The initial section will introduce the fundamental properties of HEAs, with the aim of enhancing comprehension of HEAs. Subsequently, we will present innovative synthesis methods for nanoscale HEAs, including the design principles and frequently employed techniques. The comprehensive understanding of these synthesis methods and the formation mechanisms of HEAs is crucial for further expanding the targeted development of high-entropy materials. Then, a comprehensive overview of recent advancements in the multi-field applications of HEAs, including electromagnetic absorption, energy storage, gas sensing, biomedicine, and catalysis applications. Finally, we will summarize the latest research developments in HEAs and analyze the current challenges and future prospects. This review aims to offer a thorough and insightful analysis of the current state of HEA research, and hope to inspire further exploration and development in this field.

## Fundamental Properties of HEAs

**Fig. 2:**
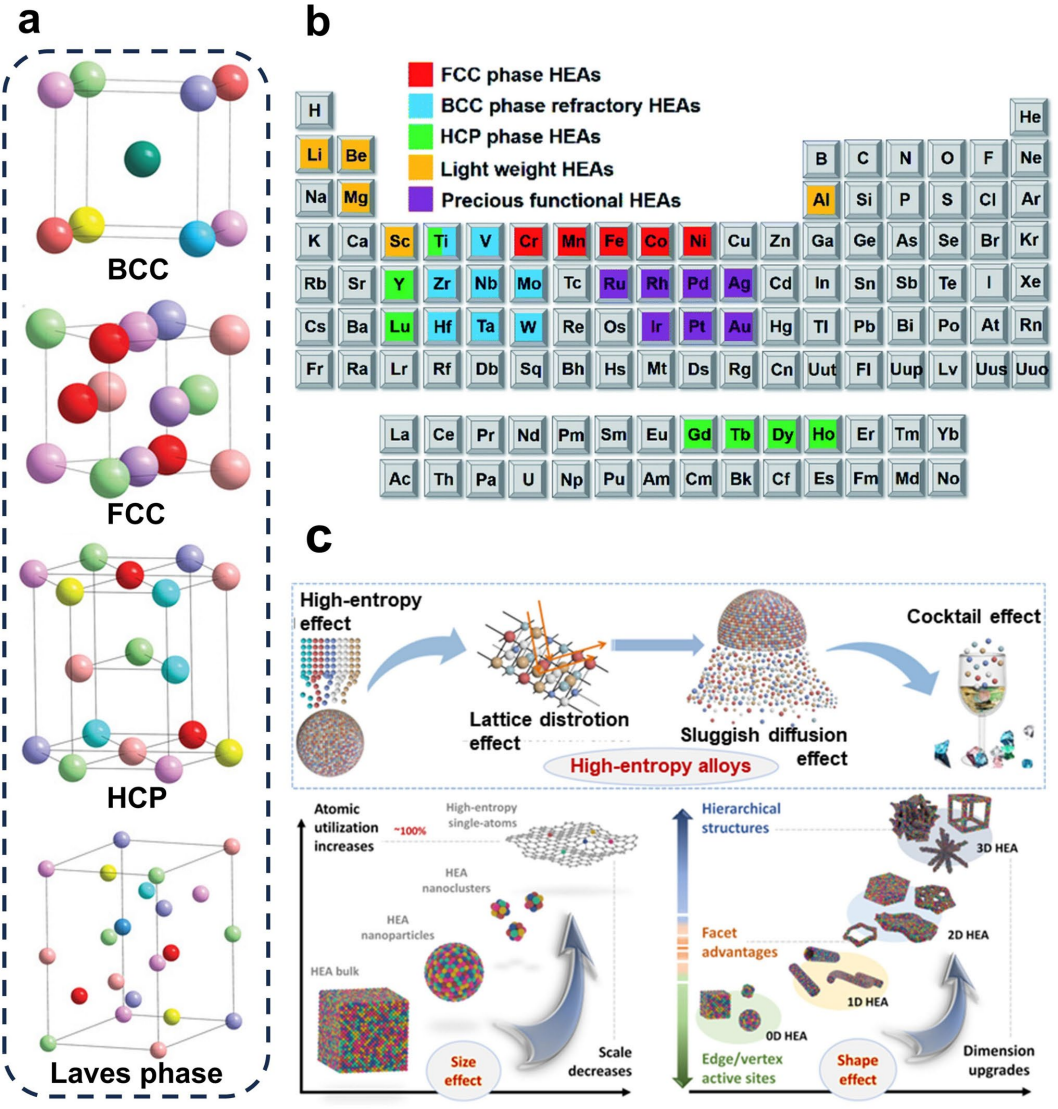
**a** Schematic illustration of the overall crystal structure for HEAs. Reproduced with permission [46]. Copyright 2022, Wiley. **b** Common elements in HEA and the various classifications of HEAs. Reproduced with permission from Ref [42]. Copyright 2021, Royal Society of Chemistry. **c** Four core effects and two other non-core effects of HEAs. Reproduced with permission [47]. Copyright 2024, American Association for the Advancement of Science

The formation of solid solutions in HEAs has been shown to be influenced by atomic size, crystal structure, valence, and electronegativity of the constituent elements [40]. Based on this finding and incorporating thermodynamic considerations, researchers have summarized five key factors that affect HEAs formation: mixing entropy, mixing enthalpy, atomic size, valence electron concentration, and electronegativity differences [41, 42]. Mixing entropy plays a crucial role in the formation of single-phase solid solutions in HEAs, while mixing enthalpy reflects the interactions between the constituent elements and significantly impacts the microstructure of HEAs. In contrast, atomic size, electronegativity differences, and valence electron concentration are intrinsic properties of the elements and are closely related to the phase stability, structural stacking, and phase transitions of HEAs. These factors serve as intrinsic regulators of HEA formation. The high configurational entropy of HEAs arises from their multi-element composition, leading to the formation of stable single-phase solid solutions with face-centered cubic (FCC), body-centered cubic (BCC), hexagonal close-packed (HCP), or Laves phase structures [43] (Fig. 2a). Consequently, HEAs can be defined in two ways, the first composition-based definition referring to the presence of five or more major elements, with the percentage of each element ranging from 5% to 35%. In some cases, trace elements constitute less than 5% of the total composition. The other is the entropy-based definition, and the mixing entropy of HEAs is approximated to the constitutive entropy by the following formula [44]:

1 $$\Delta S_{{{\text{mix}}}} = - R\mathop \sum \limits_{i = 1}^{n} C_{i} \ln C_{i}$$

where $R$ is the gas constant, ${C}_{i}$ is the mole fraction of the ${i}_{\text{th}}$ element, and $n$ is the total number of elements in the HEAs. From the formula, it can be inferred that mixing entropy increases with the number of constituent elements. A higher mixing entropy facilitates the random occupation of lattice positions by different elements, thereby promoting the formation of solid solutions rather than ordered or segregated phases [45]. This formula provides a reliable method for quantitatively measuring disorder and accounts for the randomness of atomic arrangements in HEAs systems. Based on this, mixing entropy values of 1.5R and 1.0R are commonly used as the basis for classifying HEAs, medium-entropy alloys (MEAs), and low-entropy alloys (LEAs). The elements commonly employed in the synthesis of HEAs are presented in Fig. 2b.

From the definition of high-entropy, ${\Delta S}_{\text{mix}}$ is only related to the number of constituents, and according to the Gibbs free energy formula, there is a competition between the additional $\Delta {H}_{\text{mix}}$ required to mix the constituents and the increased ${\Delta S}_{\text{mix}}$ [12]:

2 $$\Delta G_{{{\text{mix}}}} = \Delta H_{{{\text{mix}}}} - T\Delta S_{{{\text{mix}}}}$$

where $T$ represents the temperature and $\Delta {H}_{\text{mix}}$ denotes the enthalpy of mixing. Entropic stability in the crystal structure is established when $T\Delta {S}_{mix}$ equilibrates with or exceed $\Delta {H}_{\text{mix}}$. The entropic stability of the crystal structure is ensured when $T\Delta {S}_{\text{mix}}$ is greater than $\Delta {H}_{\text{mix}}$. The stability of the compound increases with ${\Delta S}_{\text{mix}}$ when the negative value of ${\Delta G}_{\text{mix}}$ is large. When the system reaches a certain equilibrium value, the contribution of ${\Delta S}_{\text{mix}}$ will exceed that of $\Delta {H}_{\text{mix}}$ and should not be ignored.

### High-Entropy Effect

The high-entropy effect (Fig. 2c), initially proposed by Yeh et al*.* [6], refers to the phenomenon wherein alloys containing five or more elements in equal or nearly equal proportions exhibit a high degree of mixing entropy and are more likely to form single-phase solid solutions than intermetallic compounds. According to the classical Gibbs phase rule, the formation of intermetallic compounds depends on the number of elemental species present in the alloys. Consequently, as the number of elemental species increases, the number of phases of intermetallic compounds also rises, leading to increased structural complexity and a corresponding impact on the properties of the alloys.

3 $$P = C + 1 - F$$

where $P$ is the number of phases, $C$ is the number of elements, and $F$ is the degree of freedom in the alloy system. Most HEAs tend to form a single-phase solid solution with a uniform structure, which is significantly fewer than the number of phases predicted by classical Gibbs phase rule calculations. According to the equation for $\Delta {S}_{\text{mix}}$, an increased number of constituent elements in HEAs results in higher mixing entropy within the system. This elevated $\Delta {S}_{\text{mix}}$ reduces the $\Delta {G}_{\text{mix}}$, equilibrates the influence of $\Delta {H}_{\text{mix}}$, enhances system stability, and decreases the number of equilibrium phases of HEAs, thereby promoting the formation of single-phase solid solutions. Furthermore, higher $\Delta {S}_{\text{mix}}$ can overcome the barriers to multi-element miscibility in HEAs, leading to superior properties and enhanced thermodynamic stability. Consequently, the high-entropy effect effectively explains why the phase number of HEAs is much less than that predicted by theoretical equations.

### Lattice Distortion Effect

The lattice distortion effects observed in HEAs can be attributed to atomic size differences among the constituent elements [48, 49] (Fig. 2c). HEAs comprise multiple elements, and metal atoms of different types randomly occupy the same lattice, resulting in a highly disordered atomic arrangement. Variations in atomic size inevitably lead to deviations in the lattice matrix from ideal positions, causing significant lattice distortion. Furthermore, differences in crystal structure and bonding energy between the constituent elements also contribute to lattice distortion. The diverse atomic sizes in HEAs induce variations in local strain fields and lattice distortions, which significantly affect the mechanical and thermoelectric properties of the materials. The lattice distortion effect can enhance the hardness and thermoelectric properties of HEAs while also optimizing their chemical properties [48]. Moreover, the mismatch of atomic sizes drives HEAs into a thermodynamically non-equilibrium state, which increases the potential energy of the alloys and lowers the energy barrier for catalytic reactions, thereby enhancing their catalytic activity. Additionally, lattice distortion leads to the formation of grain boundaries, dislocations, and other defects, significantly impacting the strength, ductility, and fracture behavior of HEAs.

### Sluggish Diffusion Effect

Sluggish diffusion effect refers to the phenomenon wherein the migration and diffusion rates of atoms in HEAs are markedly slower than those observed in conventional binary or ternary alloys. This effect arises from two primary factors. First, the multicomponent composition of HEAs leads to each lattice position being randomly occupied by different neighboring metal atoms (Fig. 2c). During atomic diffusion or phase transitions, atoms tend to migrate into vacancies that are widely available in thermally equilibrated crystals above absolute zero. However, the energy levels of neighboring vacancies are not identical to those of the original lattice positions. When an atom migrates to a low-energy vacancy, it may become trapped, making it difficult for the atom to migrate out of that vacancy. Conversely, if the atom migrates to a high-energy vacancy, it may find itself unable to migrate further and might instead return to its original position. Consequently, atoms migrate to either low-energy vacancies or high-energy vacancies, which results in a decrease in their migration diffusion rate [50]. The significant energy fluctuations between microscopic lattice sites result in a substantial number of low-energy vacancies acting as potential traps, thereby impeding atomic diffusion and contributing to the sluggish diffusion effect.

In addition, the disparity in diffusion rates among the constituent elements in HEAs is a significant factor contributing to the sluggish diffusion effect. The diffusion behavior of HEAs results from the synergistic interactions of a multitude of different atoms within the alloy. The resistance to vacancy diffusion is generally greater for inert elements compared to active elements, which limits the overall diffusion and phase transition rates of HEAs. The slow migration of certain elements impedes the overall transformation process. For instance, grain growth requires the cooperative action of all constituent elements, collectively facilitating resonant migration at the grain boundaries and thereby creating the necessary conditions for growth. Notably, the formation of new phases necessitates the reorganization and redistribution of the constituent elements to achieve the required compositional conditions. In such cases, the elements with sluggish diffusion characteristics play a crucial role, constraining the overall rate of transformation. Simultaneously, the sluggish diffusion process endows HEAs with enhanced phase stability, increased creep resistance, and elevated high-temperature strength, thereby maintaining the exceptional cyclic stability of HEAs during catalytic processes. Moreover, the sluggish diffusion process allows for the precise modulation of the material's microstructure, significantly enhancing its properties.

### Cocktail Effect

In 2015, Ranganathan [51] first introduced the polymetallic cocktail effect, emphasizing the significance of multi-element composition in alloy development. The cocktail effect of HEAs refers to the unexpected synergies that arise when the constituent elements are mixed (Fig. 2c). The performance of HEAs is not solely dictated by the individual properties of each element; rather, it is also influenced by the interactions among multiple elements. Consequently, the combination of various components may lead to the emergence of unique properties that are absent in single-element systems. Generally, the synergistic mixing of multiple components in HEAs not only produces unpredictable results but can also yield outcomes that exceed the cumulative effects of the individual elements. Unlike the other core effects previously discussed, the cocktail effect is an assumption that does not require empirical evidence. Nevertheless, it serves as a reminder to researchers that unique material properties may arise from the unexpected synergies between multiple elements. The cocktail effect is predicated on the notion that the properties of a given material are inherently unpredictable and are significantly influenced by synergistic interactions among its constituent elements. These interactions can manifest in various ways, including the emergence of specific combinations of physical properties (such as the very low expansion coefficient), catalytic response properties (such as the photovoltaic conversion), and structural properties (such as the combination of high strength and toughness, or the combination of fatigue resistance and ductility) [52, 53]. The properties of materials are closely linked to a multitude of factors, including composition, microstructure, and electron distribution. The cocktail effect underscores the importance of remaining open to nonlinear outcomes and unforeseen properties that may emerge from the unconventional combinations inherent in the expansive compositional range and intricate microstructure of HEAs.

### Other Non-core Effects

*Size effect* Conventional bulk HEAs often face limitations in their application across multi-fields due to their large size, which restricts the complete exposure of active atoms on their surfaces. Reducing the size to the nanoscale or even atomic level can significantly enhance the exposure of active atoms, and offers unlimited possibilities for the application of HEAs in the fields of electrocatalysis [54], energy storage [55] and biomedicine [56] (Fig. 2c). Notably, as the size of HEAs decreases, the exposure of surface atoms increases exponentially, providing a rich surface area and a greater number of unsaturated coordinated surface atoms, which facilitates the formation of more active sites. Furthermore, similar to conventional nanomaterials, nanoscale HEAs exhibit distinct electronic energy levels, specific surface interfacial effects, quantum size effects, and quantum tunneling effects [57]. The size effect of HEAs indicates that their performance in relevant applications is closely correlated with their dimensions. For instance, the catalytic performance of HEAs can either increase or decrease depending on the reduction in nanoparticle size during electrocatalytic reactions.

*Shape effect* Zero-dimensional (0D) nanomaterials are the most thermodynamically stable and the easiest to synthesize, making 0D nanoparticles (NPs) the most common form of current HEAs. However, 0D HEAs typically exhibit low activity due to their limited surface-active sites and uncontrolled surface electronic structures [58]. Nanomaterials with anisotropic properties, on the other hand, can provide additional anchor points for carriers, enabling the exhibition of unique properties. By developing HEAs into these specific anisotropic structures, more low-coordinated edge atoms can be exposed to the reaction system, thereby enhancing performance. Huang et al*.* [59] developed a template- directed method for the programmable synthesis of various HEAs, including 0D nanoparticles, 1D nanowires, 2D nanorings, and 3D nanodendrites, which were subsequently applied to ethanol oxidation reactions. Notably, among these structures, HEAs with 2D nanorings exhibited the highest catalytic performance, demonstrating a 25.6-fold and 16.3-fold increase in mass activity compared to commercial Pd/C and Pt/C catalysts, respectively. Furthermore, they retained a catalytic activity of 2.56 A mg^−1^_Pd+Pt_ even after a 20,000 s CA test and showed no significant decline in mass activity during a continuous 60,000 s cycling test, exhibiting its excellent durability. Thus, formulating HEAs with anisotropic structures represents a promising direction for advancing their development (Fig. 2c). In conclusion, the multidimensional evolution of HEAs from 0D nanoparticles to 1D nanowires, 2D nanosheets, and even 3D nanocubes and cubic frameworks represents a significant strategy for enhancing their physicochemical properties and expanding their multidisciplinary applications.

## Synthesis Strategies of Nanoscale HEAs

To enable the intermixing of metals with disparate chemical properties, HEAs are frequently synthesized through thermodynamic non-equilibrium processes that employ high temperatures and pressures to achieve that states. This method facilitates the miscibility of elements through the application of high temperatures and pressures, thereby enabling the expeditious molding of the material through the rapid cooling process. This approach prevents atomic diffusion, facilitating the formation of multiple elements in metastable alloys. The synthesis of nanoscale HEAs presents a significant challenge compared to traditional methods for the synthesis of bulk HEAs. The kinetic and thermodynamic considerations involved in the homogeneous mixing of multiple elements with disparate physicochemical properties and the formation of a single-phase solid solution are inherently complex [60]. Additionally, the migration and aggregation phenomena that occur at high temperatures present a significant challenge to maintaining the uniformity and structural stability of HEAs during the synthesis process. Using a thermodynamically induced method, Li et al*.* [61] applied an ultra-high heating rate (3,000 K s^−1^) to achieve rapid melting and homogeneous mixing of HEA precursor ions, followed by an extremely fast cooling rate (2,400 K s^−1^) to prevent inter-ion migration and aggregation, completing the entire process within 1200 ms. Therefore, It is essential to conduct a comprehensive analysis of the synthesis methods and conditions employed in the production of nanoscale HEAs, in order to ensure precise control over the compositional distribution and morphology of the resulting nanostructured HEAs.

Over the past decade, researchers have developed a multitude of synthetic strategies for preparing HEAs. These strategies are broadly categorized into three main approaches: solid-state, liquid, and gaseous synthesis [62]. Mechanical alloying is the most common solid-state synthesis technique, which offer a straightforward, cost-effective approach to large-scale HEA synthesis. However, due to its high-energy consumption and susceptibility to oxidation, achieving sufficient homogeneity is often challenging, requiring subsequent pressing and sintering [63]. The second approach is liquid synthesis, which includes various techniques such as laser melting. Although this method is commonly employed for the synthesis of HEAs, it typically necessitates relatively high temperatures to ensure the requisite homogeneity of the mixing and fusion of the constituent elements. Additionally, this method often produces dendritic microstructures that require extensive heat treatment to achieve homogenization [64]. Furthermore, the arc discharge method has garnered significant attention from researchers. This technique primarily utilizes the arc generated between electrodes as a heat source to melt metals, which subsequently solidify into HEAs under vacuum or an inert atmosphere [65, 66]. Zhang et al*.* [67, 68] successfully synthesized a series of HEAs with high photothermal conversion efficiency across the entire solar spectrum (250 to 2,500 nm) using arc discharge, including septenary HEAs (FeCoNiTiVCrCu) and HEAs with 21 super-mixed elements (FeCoNiCrYTiVCuAlNbMoTaWZnCdPbBiAgInMnSn), which were reported for the first time. It is important to note that it overcomes the immiscibility of the 21 elements through the high-entropy effect and a non-equilibrium synthesis process. In this process, the introduction of vapor pressure balances the evaporation rates of the composite elements, playing a crucial role in promoting their homogeneous mixing. Remarkably, the synthetic method achieved unprecedented success in synthesizing 21-element ultra-mixed HEAs from a strongly repulsive system, which was never previously reported. This work offers new insights and approaches for the discovery and application of novel HEAs. Finally, there are gas-phase synthesis methods, which mainly include atomic layer deposition, magnetron sputtering, and vapor phase deposition. These methods typically require sophisticated and expensive equipment, making the synthesis process more complex [69]. Given the wide range of traditional and modern techniques for synthesizing HEAs, developing innovative methods to produce HEAs with unique properties remains essential.

Novel design and synthesis methods for HEAs have been explored to optimize their performance across various fields. As research advances, there is a growing demand for customizing HEAs to meet the specific requirements of various applications. Additionally, synthesizing nanoscale HEAs poses significant challenges, particularly in selecting constituent elements and forming stable single-phase solid solutions during simultaneous reduction. For example, Furukawa et al*.* [70] developed a novel high-entropy catalyst by incorporating less active transition metals (Co and Cu) and typical inert metals (Ga and Sn) to partially replace highly active Pt and Ge sites. The multi-metallic design of PtGe, leveraging site isolation and entropy effects (k_d_^−1^ = *τ* = 4146 h = 173 d), effectively suppressed side reactions and enhanced catalytic stability, which can work in propane dehydrogenation at 600 °C for at least 2 months. Therefore, understanding the design principles and growth mechanisms of HEAs is crucial to overcoming the challenges associated with nanoscale HEAs synthesis. In the following sections will first discuss the design principles of HEAs, with a specific focus on nanoscale synthesis methods, then analyze the corresponding growth mechanisms and the precise control of their structures.

### Design Strategies for Nanoscale HEAs

**Fig. 3:**
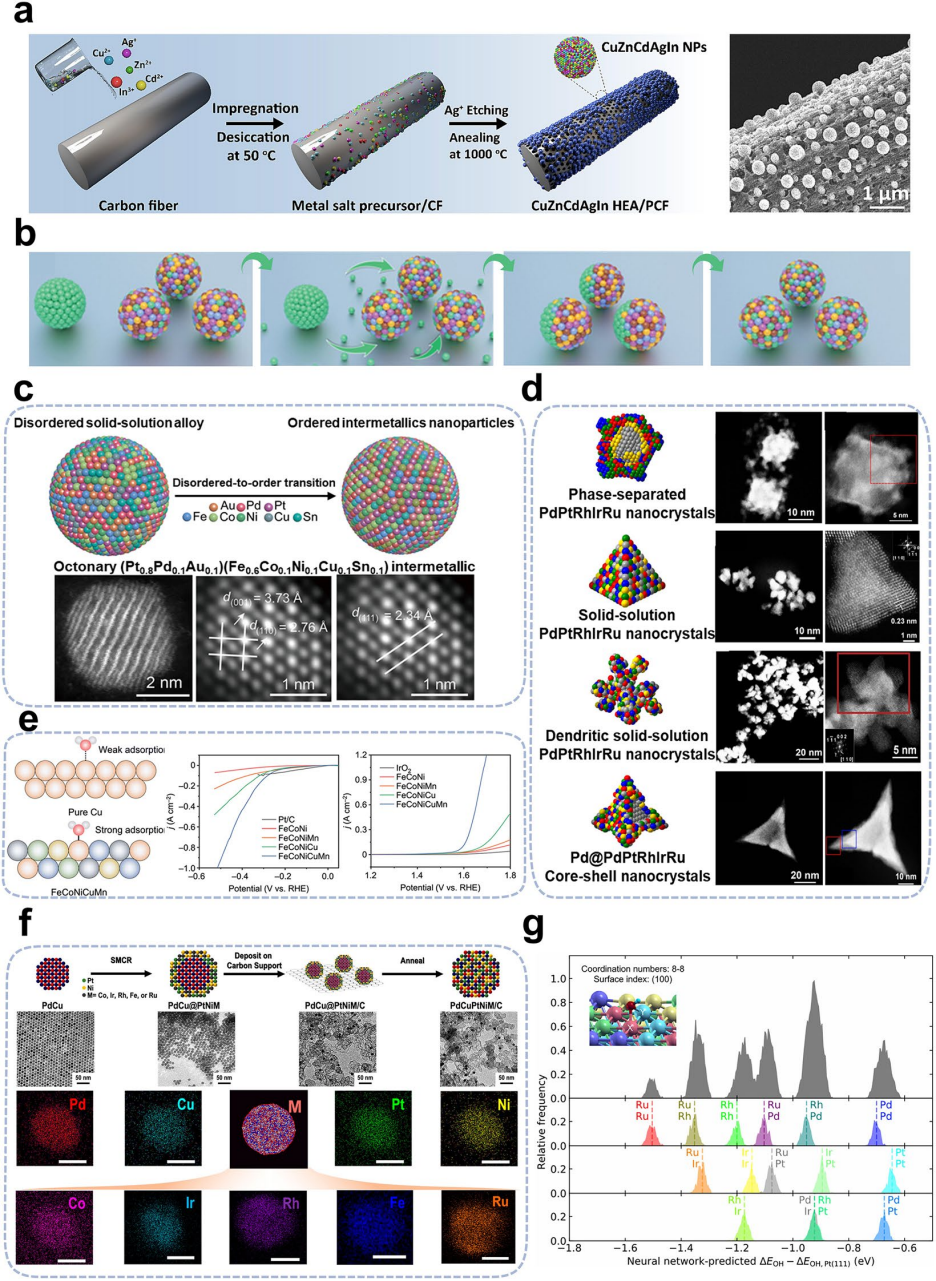
**a** Synthesis schematic illustration of HEAs and their corresponding transmission electron microscopy images distributions. Reproduced with permission [78]. Copyright 2023, Elsevier. **b** Schematic illustration depicts the thermodynamically driven solid-phase diffusion of gold nanoparticles in HEAs@C. Reproduced with permission [79]. Copyright 2023, Wiley. **c** Schematic diagram illustrates the phase transition of HEAs from a disordered to an ordered state (top) and the lattice structure (bottom). Reproduced with permission [76]. Copyright 2022, American Association for the Advancement of Science. **d** Schematic diagrams of HEAs with different morphological structures (left) and corresponding transmission electron microscopy images (right). Reproduced with permission [80]. Copyright 2023, American Association for the Advancement of Science. **e** Adsorption strengths of intermediates on catalyst metal sites (left) and the polarization curves for different catalysts(right). Reproduced with permission [84]. Copyright 2023, Royal Society of Chemistry. **f** Three-step process for the preparation of HEAs (top) and corresponding transmission electron microscopy images (bottom). Reproduced with permission [85]. Copyright 2022, American Chemical Society. **g** Frequency distribution of OH ∗ adsorption on (106) surface of HEAs from theoretical modeling calculations. Reproduced with permission [87]. Copyright 2020, Elsevier

In recent years, interest in HEAs has surged across various research fields due to their unique compositional properties and exceptional catalytic activity. To synthesize HEAs with high activity and stability, rational design principles offer valuable guidance for future research. The formation of single-phase solid-solution HEAs is generally governed by the following principles: (1) atomic radius differences between elements should not exceed 6.6%, and (2) mixing enthalpy should range between −11.6 and 3.2 kJ mol^−1^. In some cases, entropy-enthalpy ratios of at least 1.1 may also be considered [71, 72]. To form HEAs in a single-phase solid solution, the elemental composition must meet these criteria. Additionally, rational design methods can significantly improve the atomic utilization efficiency of HEAs. This can be achieved by introducing porous or multilayer structures, which increase the effective contact area between atoms and reactants [73] (Fig. 3a). The intrinsic activity of the target element can also be enhanced by modifying the local coordination environment which can be achieved through doping, component modulation, and nanoparticle inter-supporting interactions [74] (Fig. 3b).

*Structure and morphology control* The crystal structure of HEAs plays a crucial role in determining their catalytic activity. Different crystal structures alter the electronic structure, which generates varying active sites. This significantly impacts the adsorption and reaction kinetics of reactant molecules [75]. Phase structure can primarily be manipulated through chemical and physical modifications. Chemical modifications are governed by five key parameters: mixing entropy, mixing enthalpy, elemental electronegativity difference, valence electron concentration, and atomic size difference. Physical modifications influence phase structure primarily through variables such as temperature, pressure, and magnetic field. Annealing after high-temperature heating has been shown to promote interatomic diffusion and the formation of ordered high-entropy intermetallic compounds. Hu et al*.* [76] described a method for inducing a disorder-to-order phase transition in nanoparticles, where rapid heating followed by rapid cooling preserves the resulting high-entropy intermetallic structure. This transition produces phase-stabilized nanoscale high-entropy intermetallic compounds (Fig. 3c). Luo et al*.* [77] developed FeCoNiCuPd HEA NPs exhibiting two distinct phases by combining ligand-assisted interfacial assembly and annealing under an NH_3_ atmosphere. The precise structural design enabled the creation of HEAs with stable, chemically ordered phases, excellent properties, and advanced mesoporous functionality.

Furthermore, the surface morphology of the material is primarily determined by the relative balance between the deposition rate and the surface diffusion rate of reactive phase atoms. This can be controlled by adjusting the concentration and drop acceleration rate of the reactive phase reagents, as well as the solvent phase type (Fig. 3d). Yang et al*.* [80, 81] proposed an effective strategy for the controlled synthesis of platinum-group five-element HEAs nanocrystals. Through the analysis of reduction kinetics and mixing entropy during HEAs formation, and by controlling the quantity of each metal precursor and injection rate, the synthesis of typical solid-solution HEAs, dendritic solid-solution HEAs, and core–shell HEAs can be predictably and controllably achieved. In light of the preceding findings, Yang et al*.* subsequently presented ten distinct Fe-group metal (selection of three elements) and Pt-group metal (selection of two elements) co-composed quintuple HEAs, which exhibits a square atomic arrangement and possess the capacity to precisely regulate the distribution of each constituent element. The PtRuFeCoNi HEAs exhibit remarkable catalytic activity and durability, outperforming commercial Pt/C catalysts.

*Element selection* The chemical composition and element selection of HEAs are critical in determining their properties. In the compositional design of HEAs, the high-entropy mixing of multiple elements is crucial, as it is fundamental to the special properties of HEAs. Additionally, the concentration of each element and the state of mixing are vital to the performance of HEAs. The overall performance of HEAs can be optimized by adjusting the specific state of each component. The intrinsic properties of HEAs are primarily determined by their elemental composition. Extensive research has been conducted into various metal combinations and optimal compositions with specific properties. Zhu et al*.* [82] developed a FeCoNiXRu HEAs system with varied active sites to explore the contribution of different elements, where X can be substituted with Cu, Cr, or Mn. Differences in electronegativity between mixed elements induce significant charge redistribution, creating highly active Co and Ru sites with optimized energy barriers. The findings indicate that the catalytic activity of HEAs can be enhanced by modulating the electronegativity of individual components, thereby optimizing the overall synergistic and electronic properties.

Additionally, the number of elements in the alloy can be adjusted to optimize the synergistic interactions between different sites, which is crucial for achieving high catalytic activity and selectivity in HEAs. Kitagawa et al*.* [83] conducted a comprehensive comparative analysis of the ethanol oxidation reactivity of ternary (PdPtRh), quaternary (IrPdPtRh), penta- (IrPdPtRhRu), and hexa- (RhRuPdOsIrPt) HEAs. Their findings demonstrated a significant increase in catalytic activity with the addition of components, further highlighting the importance of synergistic effects among multiple elements. Moreover, achieving optimal performance requires a rational selection of elements when designing HEAs. However, traditional experimental screening methods are often time-consuming and costly. Thus, combining theoretical calculations with experimental data from specific reaction systems allows for the rapid and large-scale screening of elemental compositions. Wang et al*.* [84] proposed a strategy to regulate high-entropy atomic environments by combining density functional theory (DFT) calculations with experimental results. The low-electronegativity element Mn was combined with the high-electronegativity elements Cu, Fe, Co, and Ni to form HEAs. This design aimed to drive inactive Cu atoms to electron-rich active sites, converting inactive regions into electrocatalytically active sites and enhancing overall catalytic activity (Fig. 3e). Compared to commercial Pt/C catalysts, which require 302 mV to achieve a current density of 100 mA cm^−2^, FeCoNiCuMn HEAs require only 281 mV of overpotential to reach the same current density. Furthermore, electrocatalytic kinetics studies revealed that HEAs exhibit a lower Tafel slope (53 mV dec^−1^) and follow the Volmer-Heyrovsky mechanism in alkaline electrolytes.

*Surface modification* Material surfaces are critical sites for numerous chemical reactions and physical transformations, and serve a significant function in a multitude of applications. The design and optimization of HEAs through surface engineering present an effective approach to enhance their performance. The surface of HEAs can be modified or endowed with specific functionalities, such as targeting, antioxidant properties, and catalytic activity, by grafting additional ligands. This modification facilitates the performance of HEAs in multi-fields by improving interfacial interactions and exposing active sites. Skrabalak et al*.* [85] developed a generalized method for synthesizing HEAs via the thermal conversion of core–shell NPs. This method begins with the synthesis of PdCu NPs, which serve as seeds. Subsequently, the PtNiM-shell (M = Fe, Co, Rh, Ru, Ir) layer is modified on the surface of the PdCu NPs to form high-entropy intermetallic compounds with a core–shell structure (Fig. 3f). This is achieved through a seed-mediated co-reduction process. Finally, the core–shell NPs are deposited on a carbon carrier and subjected to heat treatment, which promotes atomic mixing and the formation of HEAs. In addition to the deposition of metal elements, the surfaces of HEAs can also be modified using organic ligands. Liang et al*.* [20] successfully developed a generalized metal–ligand cross-linking strategy for the preparation of ultrasmall PtPdRuRhIr HEAs, which were based on aldol condensation reactions. During the cross-linking process, organic ligands were grafted onto the surface of the HEAs, resulting in materials with excellent POD-like enzyme activity and high photothermal conversion efficiency.

*Machine learning* The variable composition of HEAs offers researchers a multitude of design options. However, the vast array of elemental combinations presents significant challenges for the rapid screening and design of HEAs that exhibit both high performance and high activity. In recent years, machine learning and theoretical prediction methods have been extensively employed to forecast the surface and subsurface compositions of alloying materials, including single-atom alloys and ordered intermetallics [86]. Consequently, the application of theoretical prediction models and machine learning algorithms for the rapid screening of HEAs will assist researchers in the efficient design and synthesis of high-performance HEAs. Singh et al*.* [87] successfully predicted the surface adsorption energy of HEAs by integrating a neural network with density functional theory (DFT) calculations, taking into account both ligand effects (the spatial arrangement of different elements) and coordination effects (variations in crystallographic surfaces and defects). The results align with the reported experimental results, demonstrating the accuracy and effectiveness of the theoretical prediction methods in HEAs design (Fig. 3g). The model demonstrates high predictive accuracy, with an average absolute error of 0.09 eV, and exhibits strong generalization, making it broadly applicable to bimetallic catalysts. Additionally, the model is simple and can be further reduced to a linear scaling model with only 36 parameters. Subsequently, to enhance the precision of theoretical predictions and the depth of machine learning, Zhao et al*.* [88] developed an accurate and efficient atom graph attention (AGAT) network to accelerate the development of high-performance high-entropy electrocatalysts (HEECs). The model statistically verified the reliability of classical d-band theory and scaling relations concerning HEECs' surfaces. Additionally, it was shown that HEECs can effectively circumvent the scaling relations by providing sufficiently versatile local environments. This work offers a novel approach for the rapid high-throughput screening and rational design of high-performance HEECs.

### Electrochemical Synthesis

**Fig. 4:**
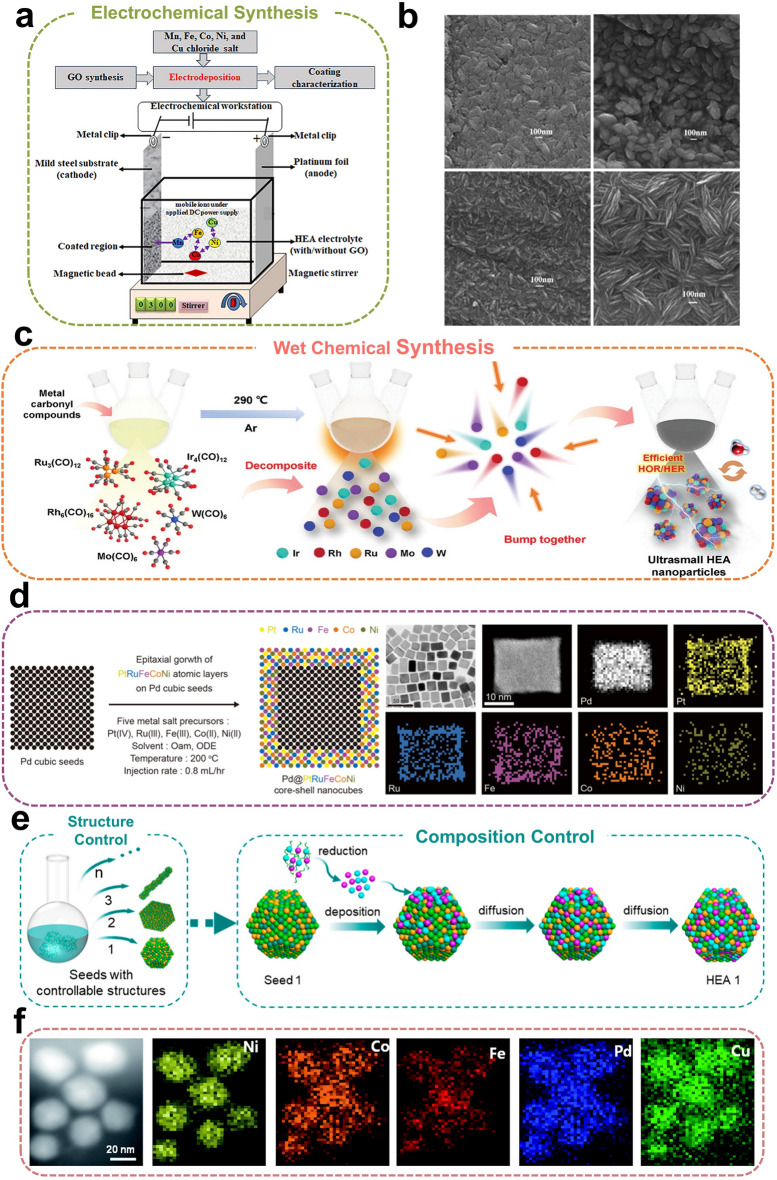
**a** Schematic diagram illustrates the fundamental principle of electrodeposition devices. Reproduced with permission [96]. Copyright 2022, Elsevier. **b** Thin films of BiFeCoNiMn HEAs obtained via electrodeposition at different elemental molar ratios. Reproduced with permission [90]. Copyright 2008, Elsevier. **c** Schematic illustration of the wet chemical synthesis of IrRuRhMoW HEAs. Reproduced with permission [97]. Copyright 2024, Wiley. **d** Schematic of Pd@PtRuFeCoNi HEAs atomic layer catalysts (left) and the elemental mapping images (right). Reproduced with permission [81]. Copyright 2024, American Association for the Advancement of Science. **e** Schematic synthesis of HEAs with different morphology and structure. Reproduced with permission [59]. Copyright 2023, American Chemical Society. **f** Elemental mapping images for PdFeCoNiCu HEAs. Reproduced with permission [94]. Copyright 2021, Royal Society of Chemistry

Electrochemical deposition is an effective method for the growth and preparation of metal nanoparticles (Fig. 4a). Moreover, this method allows precise control over the nucleation and growth of NPs. Additionally, the compositional state of the nanoparticles can be easily modified by adjusting relevant parameters. In this approach, the redox reactions occurring at the electrode surface within the electrolyte are strongly influenced by the externally applied electric field. Furthermore, the composition of the nanoparticles can be precisely controlled by adjusting the voltage of the applied electric field, current density, and electrolyte composition [89]. This method, which involves the reduction of metal ions in an electrolyzer, is relatively inexpensive and operates under mild conditions. However, it requires precise control of electrolysis parameters to ensure the uniformity and purity of the alloy. The electrochemical deposition process allows the simultaneous reduction of multiple metal ions, providing a promising route for synthesizing HEAs.

In a pioneering study, Tong et al*.* [90] demonstrated a simple and effective method for synthesizing BiFeCoNiMn HEA films via electrochemical deposition. Through constant potential deposition in an organic system, they successfully synthesized BiFeCoNiMn nanorod HEAs. Subsequent research investigated the influence of electrodeposition potential and elemental molar ratios in the organic system on the formation of HEAs (Fig. 4b). Schwandt et al*.* [91] were the first to report that HEAs can also be synthesized electrochemically in a molten salt system. Premixed solid metal oxides were cathodically polarized in molten CaCl₂ electrolyte at 1,173 K, producing equiatomic refractory alloys TiNbTaZr and TiNbTaZrHf through a one-step alloying process. Compared to direct electrodeposition, pulsed electrodeposition offers greater precision in current distribution and mass transfer, enabling more accurate modulation of HEAs microstructure and chemical composition. Yoosefan et al*.* [92, 93] synthesized CoCrFeMnNi HEAs films using pulsed electrochemical deposition. In a subsequent study, they investigated the impact of pulsed electrodeposition parameters (pulse frequency and duty cycle) on the wettability and corrosion resistance of the films, and ultimately found that a pulse potential with a frequency of 2,500 Hz and a duty cycle of 60% resulted in the optimal corrosion resistance of the CoCrFeMnNi HEA films.

### Wet Chemical Synthesis

Wet chemical synthesis involves introducing a series of precursor ions into a solvent to convert metal precursors into HEAs, offering a promising approach for HEA synthesis (Fig. 4c). A key advantage of this method is that HEAs growth can be regulated by controlling parameters such as reaction temperature, solvent type, and reaction time, enabling precise control over the synthesis process. Yang et al*.* [80, 81] investigated the reduction kinetics and mixing entropy changes of five platinum-group metal precursor ions in ethylene glycol solvents under varying dropwise addition rates. Based on these findings, they developed a predictive design method for synthesizing HEAs nanocrystals. By controlling the droplet synthesis, the content of each metal precursor ion was regulated at the same or different times during the process, enabling the controlled and predictable synthesis of targeted HEAs nanocrystals, such as typical PdPtRhIrRu HEAs, dendritic PdPtRhIrRu HEAs, and Pd@PdPtRhIrRu core–shell nanocrystals. Furthermore, they developed a series of HEAs atomic layer catalysts composed of three iron-group elements and two platinum-group elements, with precisely controlled square atomic arrangements (Fig. 4d).

The precise synthesis of ultrasmall HEAs with controllable morphology and tunable composition is essential for achieving advanced performance. Wang et al*.* [94] successfully synthesized Pd-based HEAs (PdFeCoNiCu) with a small particle size (34 nm) for the first time in an atmospheric-pressure oil phase at low temperatures (≤ 250 °C) (Fig. 4f). The oil-phase solvent, oleylamine, played a crucial role in coordinating and incorporating different metal ions during synthesis. Notably, the co-reduction of metal precursor ions at 220 °C successfully synthesized HEAs with a homogeneous solid-solution structure, offering a novel approach and opening new direction for the facile synthesis of colloidal alloy materials. This co-reduction strategy not only ensures the uniform distribution of multiple elements at the nanoscale, enhancing the stability and catalytic activity of HEAs, but also enables precise control over the metal precursor ion ratio, thereby regulating the composition and structure of HEAs. The HEAs prepared using this method exhibited a mass activity of 6.51 A mg_Pd_^−1^ at 0.07 V. Their high catalytic activity was attributed to Fe-enhanced d-d electronic coupling, which compensates for the electrons on the Co site, thereby facilitating hydrolysis. Electron-rich Cu promotes overall electron transfer, while neighboring Ni contributes to the stabilization of OH. Moreover, Schaak et al*.* [95] systematically synthesized colloidal HEAs NPs and elucidated the reaction pathways. The preparation of HEAs involved the gradual reaction of a solution containing metal precursor ions, introduced into a mixture of oleylamine and octadecene at 275 °C. By altering the concentration ratio of precursor ions, tunable elemental compositions were achieved. Huang et al*.* [59] developed a template-guided synthesis approach that allows for independent control of HEAs morphology and composition, enabling the programmable synthesis of nanoscale HEAs with precisely defined structures and compositions. This method enables the synthesis of various morphologically controlled HEAs, including 0D nanoparticles, 1D nanowires, 2D ultrathin nanorings, and 3D nanodendrites (Fig. 4e). Similarly, Xia et al*.* [98] demonstrated the superior performance and stability of a novel NiCoFePtRh ultrasmall HEAs as an advanced electrocatalyst in acidic media, exhibiting the smallest particle size reported to date at just 1.68 nm. These ultrasmall HEAs exhibit an ultra-high TOF of 30.1 s⁻^1^ and optimal stability at 50 mV overpotential, showing no performance degradation after 10,000 cycles. Theoretical calculations further revealed that the actual active site, tunable electronic structure, and synergistic interactions among the five elements collectively enhanced the hydrogen evolution reaction (HER) activity of the ultrasmall HEAs. It is worth noting that Gao et al*.* [99] developed a novel non-equilibrium wet chemical synthesis method, utilizing highly reactive H species generated by DMF dehydrogenation to create a localized non-equilibrium reducing environment within the precursor seeds. This approach enables the uniform hydrothermal synthesis of HEAs with precisely controlled sizes ranging from 1 to 4 nm at 170 °C, achieving an unprecedented level of synthesis accuracy.

### Combustion-assisted Synthesis

Compared to conventional sintering, combustion-assisted synthesis offers a more economical and accessible approach to preparing HEAs. This method facilitates the formation of HEAs by introducing a combustion agent into the reaction system and utilizing the heat released during the combustion reaction. The advantage of this approach is its fast-heating rate, allowing for the synthesis of high-quality alloys in a short period of time. Moreover, this method relies on a highly exothermic aluminothermic reaction, which is a notable contrast to the heating employed in a reactor during the conventional sintering process. Fu et al*.* [100] first proposed the pioneering sol–gel self-combustion technique for synthesizing CoCrCuNiAl HEAs. The resulting nanoparticles have an average size of approximately 14 nm and exhibit distinctive superparamagnetic and magnetic properties. High-temperature combustion of metal nitrate precursors initially produced (Co, Cu, Mg, Ni, Zn)O high-entropy oxides, which were subsequently reduced in situ to CoCrCuNiAl HEAs in a reducing atmosphere. The experiment demonstrates the effectiveness of the sol–gel self-combustion synthesis method, which is not limited by high mixing entropy. Politano et al*.* [101] pioneered the preparation of ceramic composite HEAs via combustion synthesis, in which self-sustained synthesis occurs through the release of substantial heat, propagated throughout the sample as a combustion wave. This wave melts the metal powders of the constituent elements, which then crystallize to form HEAs.

**Fig. 5:**
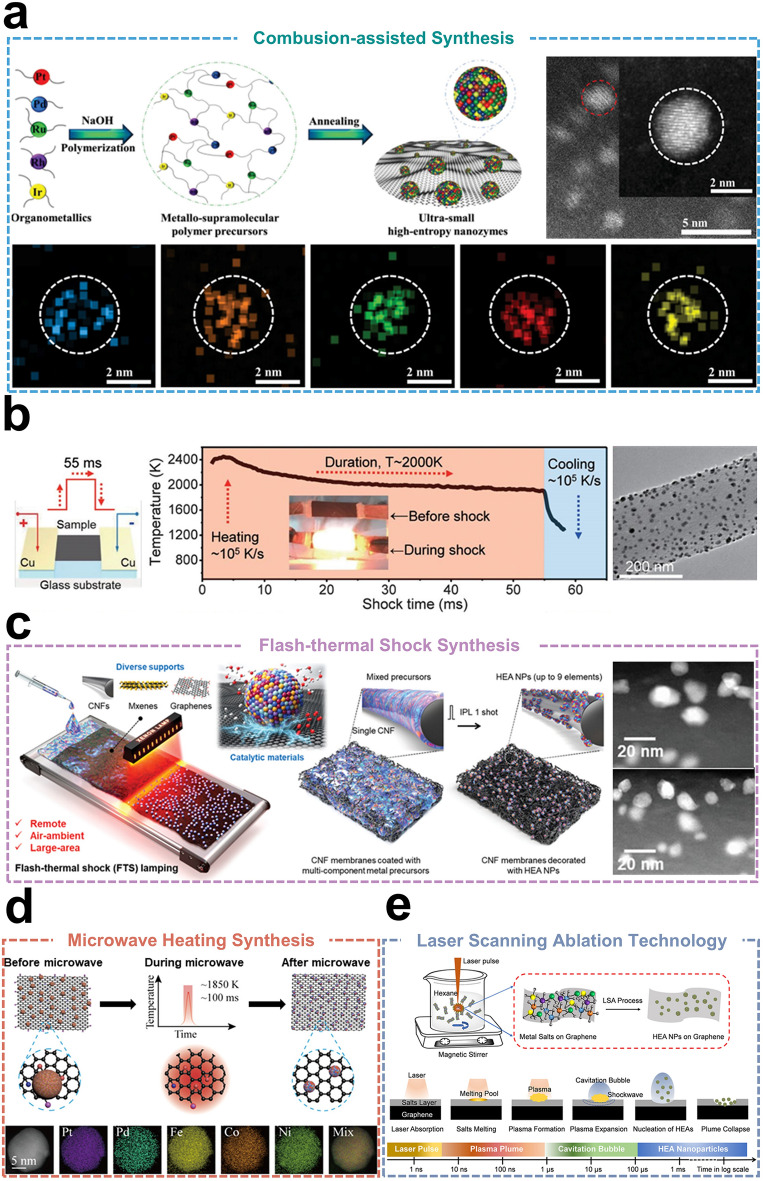
**a** Organometallic precursors polymerized and annealed to produce ultrasmall HEAs (top) and corresponding elemental mapping images (bottom). Reproduced with permission [20]. Copyright 2023, Wiley. **b** Schematic of the temperature–time evolution curve during a 55 ms thermal shock. Reproduced with permission [103]. Copyright 2018, American Association for the Advancement of Science. **c** Schematic of the method for non-contact, large-scale, ambient-air synthesis of HEAs. Reproduced with permission [23]. Copyright 2023, Wiley. **d** Schematic representation of the formation of HEAs on the surface of rGO by microwave heating. Reproduced with permission [107]. Copyright 2021, American Chemical Society. **e** Schematic representation of HEAs synthesized on graphene surface by LSA strategy. Reproduced with permission [108]. Copyright 2023, Elsevier

Liang et al*.* [20] developed an innovative approach for synthesizing ultrasmall PtPdRuRhIr HEAs using a ligand cross-linking strategy based on aldol condensation. This approach involves the equal mixing of metal precursors, followed by the formation of supramolecular polymer precursors in an alkaline environment (Fig. 5a). The reaction between organometallic compounds and acetylacetone forms carbon–carbon bonds, resulting in a homogeneous random intercalation structure. The intercalated structures were then subjected to high-temperature combustion and thermal annealing to completely remove the organic ligands, producing ultrasmall HEAs. The HEAs obtained by this strategy have a size of less than 3 nm and are anchored by carbon–carbon bonds. The HEAs demonstrate both enzyme-like catalytic activity and a high photothermal conversion effect, enabling the effective elimination of breast cancer cells. It is typically observed that HEAs comprising noble and base metals do not exhibit complete miscibility. However, Yamashita et al*.* [102] recently generated spillover hydrogen species with high reduction potentials from noble metals (Ru and Rh) by leveraging the strong spillover effect of TiO_2_. These species rapidly migrated to base metals (Ni and Cu), reducing them to form TiO_2_-supported CoNiCuRuPd HEAs during subsequent drying and thermal annealing. The TiO_2_-supported CoNiCuRuPd HEAs exhibit high catalytic activity and excellent stability during CO₂ hydrogenation reactions.

### Flash-thermal Shock Synthesis

Carbon thermal shock (CTS) synthesis involves the uniform mixing of an appropriate amount of carbon source (activated carbon or carbon black) with metal precursor salts at high temperatures, which is an innovative way to generate HEAs. When temperatures exceeding 2,000 K, the carbon source undergoes violent combustion, inducing a thermal shock that rapidly reduces the metal precursor ions to metal atoms. These atoms then melt, form a solid solution, and rapidly cool to produce HEAs. High temperatures and short durations are key factors for achieving homogeneous mixing of elements and inhibiting the agglomeration of nanoparticles. These conditions help lower the system's free energy, increase the entropy of mixing, and maintain the homogeneity of single-phase or multiphase structures. In pioneering research, Hu et al*.* [103] were the first to successfully apply CTS synthesis for alloying up to eight distinct elements into single-phase solid-solution NPs (Fig. 5b). The alloy's microstructure and properties are regulated by controlling parameters such as the matrix (carbon nanofibers), temperature (~ 2,000 K), impact duration (55 ms), and cooling rate (~ 10^5^ K s^−1^) of the CTS process. Rapid high-temperature impacts accelerate the diffusion and homogeneous distribution of metal atoms, leading to the formation of uniform alloys. While CTS synthesis ensures the structural stability and compositional homogeneity of HEAs, it demands precise temperature control during both heating and cooling. Moreover, the complex post-processing steps following thermal shock, which involve the removal of residual impurities and interfering elements, present additional challenges for the large-scale application of this method.

Achieving high-coverage alloy nanoparticles on minimally exposed carbon substrate surfaces has been a pivotal technical challenge for the CTS synthesis method. To address these challenges, Jung et al*.* [104] employed a strategy using partially carbonized cellulose as the carbon source due to its interconnected aromatic rings and numerous edge sites. This approach effectively achieved high surface coverage (~ 85%). CTS synthesis requires an electrically conductive support, and the efficiency of Joule heating depends on the quality of the carbon source, with uniform heating across large areas being the primary challenge. Considering these limitations, Choi et al*.* [23] proposed a photothermal method as an alternative to CTS, which is compatible with surrounding environments, synthesis areas, and remote processes, and does not require specific material selection (Fig. 5c). It achieves rapid and large-area heating of HEA NPs through light interactions with materials such as carbon nanofibers (CNFs), graphene oxide, and MXenes. The instantaneous high-temperature annealing process, comprising a single 20 ms flash of 4.9 J cm^−2^ on the CNFs film, resulted in the successful synthesis of HEAs containing nine elements. The non-contact light-material interactions in this method represent a significant innovation in HEAs synthesis, offering a promising strategy for large-scale production.

### Microwave Heating Synthesis

Microwave heating synthesis rapidly generates high temperatures and ensures uniform heat transfer, preventing elemental or phase separation during HEAs synthesis. This method promotes the formation of HEAs by heating the material rapidly to the desired temperature. It offers the advantages of uniform heating and a fast reaction rate, but it requires addressing the complexity of the interaction between microwaves and the material. This emerging synthesis technology offers distinct advantages, including good selectivity, volumetric heating, and enhanced sintering [105]. Since Agrawal et al*.* [106] pioneered the microwave sintering of powdered metals into fully dense products, microwave heating synthesis has gained increasing attention and has been progressively applied to assist in HEAs synthesis, overcoming limitations of traditional methods. After years of development, Hu et al*.* [107] employed graphene oxide, rich in functional group defects, as a model substrate to efficiently absorb microwaves and generate average temperatures of up to 1,850 K within seconds (Fig. 5d). The rapid ramp-up heating process was subsequently employed to successfully synthesize PtPdFeCoNi HEAs, whose constituent elements decompose almost simultaneously at rapid elevated temperatures, producing liquid metals without diffusion and mixing uniformly to form HEA NPs with particle sizes approximately 12 nm. Compared to conventional high-temperature furnace heating, microwave radiation heating is a promising method for synthesizing HEAs, offering rapid, uniform temperature elevation and suitability for carbon materials of varying sizes.

### Laser Scanning Ablation Technology

In recent years, laser scanning ablation (LSA) technology has gained prominence in the synthesis of HEAs. LSA is a physical vapor deposition process that employs localized laser heating to evaporate the surface material of bulk HEAs, resulting in the generation of a plasma composed of hot atoms, ions, and clusters. This process is initiated by irradiating the surface with a high-energy laser beam. Following a cooling phase, the plasma gradually condenses to form nanoscale HEAs. Recently, Yao et al*.* [108] developed a novel LSA strategy to synthesize electrocatalysts featuring both high activity and stability for PtIrCuNiCr HEAs (Fig. 5e). The electrocatalysts were employed as both anodes and cathodes in the catalytic process, exhibiting the lowest overpotentials of approximately 190 mV during the catalytic decomposition of water, along with high current densities of up to 10 mA cm^−2^. Furthermore, the binding energy of the intermediates involved in the catalytic process is modulated by adjusting the bonding energy of the metal bonds among the constituent elements. In a recent study, Zou et al*.* [109] developed a straightforward and universal LSA method for synthesizing ultrasmall HEA NPs. The synthesis process occurs in just 5 ns at atmospheric temperature and pressure, allowing for the production of NPs with uniform compositions of up to nine elements, despite the thermodynamic limitations of solubility among these elements. The ability to deposit HEA NPs onto various substrates is facilitated by the precision of laser pulses, which effectively localize and confine energy to the targeted area. Subsequently, the HEAs were deposited onto graphene and integrated into both the anode and cathode for overall water splitting. This approach yields current densities of up to 10 Ma cm^−2^ at a mere 1.42 V, significantly surpassing those of existing catalysts. The rapid advancement and substantial potential applications of LSA technology enable the generation of diverse functional materials through the systematic substitution of target alloy materials, substrate fixation, and other variable factors [13]. This method offers a distinctive advantage in the rapid and efficient synthesis and screening of HEA materials within this field.

### Dealloying Synthesis

The dealloying synthesis method is a widely employed technique for fabricating porous structures with high specific surface areas and has also been extensively applied in the production of nanoscale HEAs. The dealloying process synthesizes HEAs through selective corrosion, which involves the partial corrosion of multicomponent solid-solution alloys, wherein the reactive metal atoms are dissolved while the relatively inert metal atoms remain. This process primarily focuses on the selective removal of reactive components from the alloy via chemical dissolution and electrochemical methods. The former is achieved by immersing the multicomponent alloy in an acidic or alkaline solution, which initiates the chemical reaction. Additionally, electrochemical methods are employed, involving the fabrication of an electrode from the multicomponent alloy and the application of a potential or electric current to oxidize or dissolve the more reactive anodic components. This process results in the formation of a continuous nanoscale multilevel pore framework composed of metal elements that are more stable and less reactive.

**Fig. 6:**
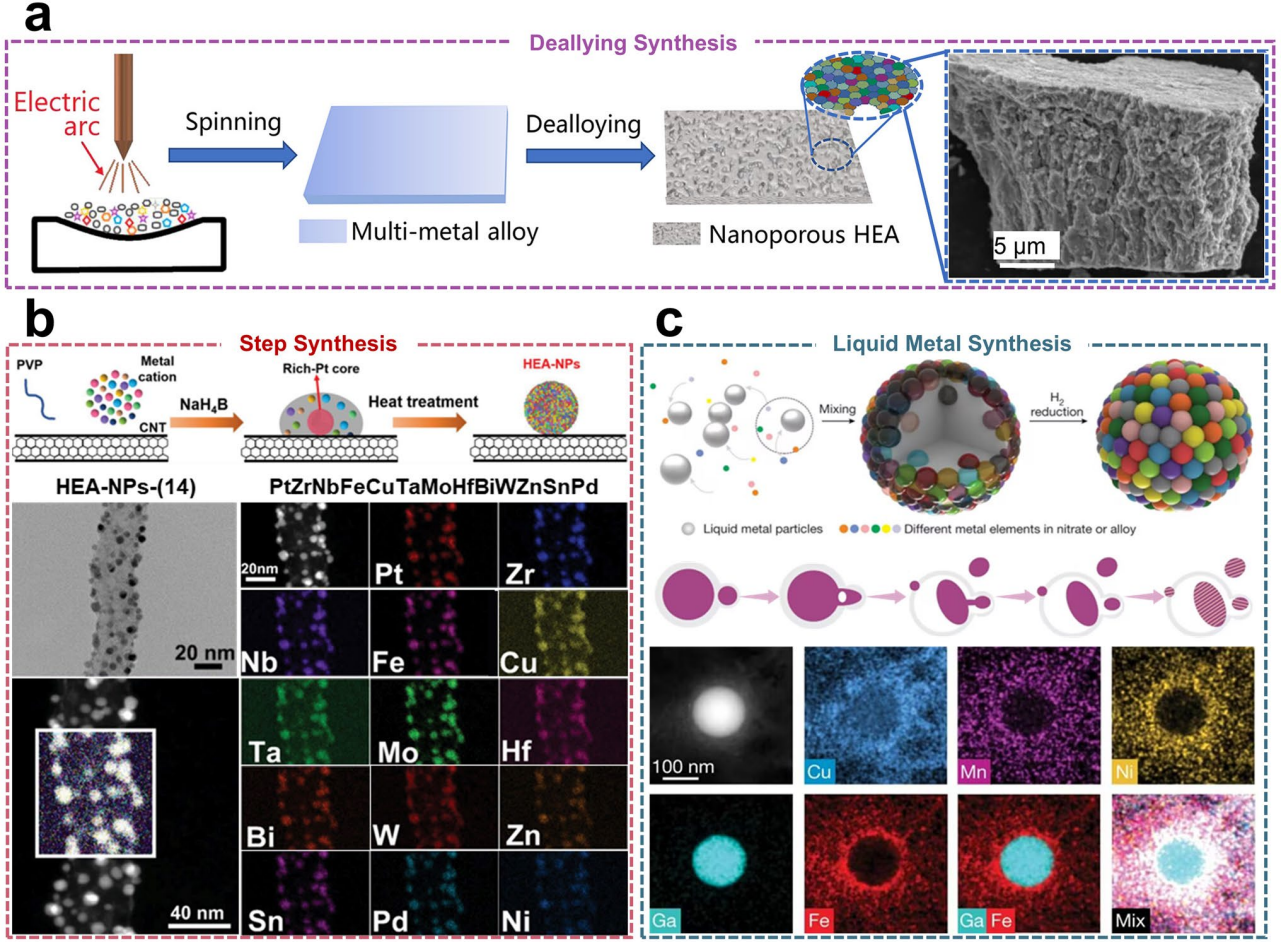
**a** Schematic diagram of the dealloying strategy for the preparation of nanoporous HEAs. Reproduced with permission [110]. Copyright 2021, American Chemical Society. **b** Schematic diagram of step alloying strategy (top) and EDS-mapping for HEA-NPs-(14) (bottom). Reproduced with permission [58]. Copyright 2023, Wiley. **c** Schematic diagram of the liquid-assisted metal synthesis process (top) and the transformation of liquid metal NPs to alloy NPs (middle). Reproduced with permission [22]. Copyright 2023, Springer Nature.

Based on the advantages of the dealloying synthesis method, Qiu et al*.* [110] reported a two-step fabrication process for synthesizing single-phase multicomponent nanoporous HEAs (Fig. 6a). These HEAs with a fine pore structure are composed of 12 (Mn_70_Ni_7.5_Cu_7.5_Co_4.2_V_4.2_Fe_2_Mo_2_Pd_0.5_Pt_0.5_Au_0.5_Ru_0.5_Ir_0.5_) or 16 (Mn_67.5_Ni_7.2_Cu_7.2_Co_4_V_4_ Fe_4_Mo_0.5_Cr_0.5_Pd_0.5_Pt_1_Au_0.5_Ru_1_Ir_0.5_Ag_0.5_Rh_0.5_Os_0.5_) elements, and a homogeneous distribution of the multicomponent elements is achieved by a one-step dealloying process in (NH_4_)_2_SO_4_ solution. Previously, there had been no precedent for the separate synthesis of nanoporous HEAs comprising up to 16 elements. The successful synthesis of the HEAs has produced materials with exceptional HER and oxygen reduction reaction (ORR) performance, exhibiting superior efficacy compared to commercial Pt/C catalysts. Additionally, these materials have demonstrated enhanced catalytic activity for the oxygen evolution reaction (OER), surpassing the performance of commercial IrO_2_ catalysts. In addition to the Mn-based HEAs previously discussed, Al is also frequently employed in dealloying synthesis methods due to its capacity for selective removal in alkaline solutions. Qiu et al*.* [111] reported a top-down dealloying strategy for the controllable stabilization of five metallic elements in a nanoscale solid phase, achieved by the alternate addition of a fifth element (Pd, V, Co, or Mn) to a predetermined set of four elements (Al, Cu, Ni, and Pt). The AlCuNiPtMn HEAs exhibit the most effective ORR catalytic activity and electrochemical durability, significantly surpassing that of commercial Pt/C catalysts. Furthermore, Liu et al*.* [112] devised a novel HEAs foil with a nanoporous structure as a multifunctional electrocatalyst through the integration of dealloying and polarization. In an alkaline environment, the HER overpotential of NiCoFeMoMn HEAs at 1,000 mA cm^−2^ is only 150 mV, and the OER overpotential at the same current density is only 350 mV. Moreover, a stable current density of 10 mA cm^−2^ can be sustained at a voltage of only 1.47 V when the alloys are utilized as either anodes or cathodes.

### Other Synthesis Methods

In conclusion, the synthesis methods for HEAs have evolved over time and have been successfully applied in various fields (Table 1). However, novel and more efficient synthesis methods are still under development. Such as the template method [59], step synthesis strategy [58], and liquid metal reaction medium [22] have also garnered significant interest from researchers. In the template method, seeds serve as a template for the morphological control of HEAs, while the reduction, deposition, and diffusion of various precursors determine the ratio of each elemental composition of HEAs [113]. By separating morphological control from component control in the synthesis of HEAs, nanoscale HEAs can be synthesized in a programmed manner [114]. The stepwise alloying process consists of two primary steps: first, the preparation of a Pt-rich core during the liquid-phase reaction, and second, the diffusion of other elemental precursors into the Pt-rich core during the subsequent heating treatment. The ΔH_mix_ of Pt with other elemental combinations is below the limitation for all elemental combinations, indicating that the preparation of Pt-rich cores facilitates the synthesis of HEAs comprising multiple immiscible elements at relatively low temperatures without phase separation (Fig. 6b). Furthermore, liquid metals can confer negative enthalpies of mixing to other elements, thereby providing stable thermodynamic conditions for the preparation of polymetallic HEAs. Notably, it also act as an ideal dynamic mixing medium (Fig. 6c).

**Table 1:** Summary of different synthesis methods for HEAs

Synthetic method	Temperature and pressure	Productivity	Size	Element applicability	References
Electrochemical synthesis	298 K / 1 atm	~ mg level	50–70 nm	Bi, Fe, Co, Ni, Mn	[90]
Electrochemical synthesis	1173 K / 1 atm	1–3 g	Porous bulk	Ti, Nb, Ta, Zr, Hf	[91]
Wet chemical synthesis	453 K / 1 atm	~ mg level	5–10 nm	Pd, Pt, Ru, Rh, Ir	[80]
Wet chemical synthesis	443 K / high pressure	~ mg level	1–4 nm	Pt, Ir, Cu, In, Ga, Pb, Ni, Co, Fe	[99]
Combustion-assisted synthesis	573 K / 1 atm	~ mg level	14 nm	Co, Cr, Cu, Ni, Al	[100]
Combustion-assisted synthesis	1073 K / 1 atm	~ mg level	1.5 nm	Pt, Pd, Ru, Rh, Ir	[20]
Flash-thermal shock synthesis	2000 K / 1 atm	~ 100 mg	3–25 nm	Pt, Pd, Co, Ni, Fe, Au, Cu, Sn, Rh, Ru, Mo, Ce	[103]
Flash-thermal shock synthesis	2073 K / 1 atm	~ mg level	< 10 nm	Pt, Pd, Ru, Ir, Fe, Co, Ni, Cu, La, Ce, In Sr	[23]
Microwave heating synthesis	1050 K / 1 atm	~ 100 mg	10–50 nm	Pt, Pd, Au, Rh, Ru, Fe, Co, Ni, Cu, Al	[107]
Laser scanning ablation technology	- / 1 atm	~ mg level	2 nm	Pt, Ir, Cu, Ni, Cr	[108]
Laser scanning ablation technology	- / 1 atm	~ 10 g	1–10 nm	Pt, Ir, Ru, Au, Co, Cr, Fe, Mn, Ni	[13]
Dealloying synthesis	- / 1 atm	~ g level	Porous bulk	Mn, Ni, Cu, Co, V, Fe, Mo, Cr, Pd Pt, Au, Ru, Ir, Ag, Rh, Os,	[110]
Dealloying synthesis	- / 1 atm	~ g level	200–300 nm	Ni, Co, Fe, Mo, Mn	[112]

Generally, each synthesis method possesses distinctive advantages and characteristics, making it crucial to conduct a comprehensive evaluation to select the most suitable method based on the desired properties [115, 116]. While the benefits of each strategy are evident, significant challenges remain. To fully capitalize on the potential of HEAs in catalysis, biomedicine, and energy storage, it is essential to advance the development of efficient, cost-effective, and precise preparation methods for HEAs.

## Multi-field Applications of Nanoscale HEAs

The unique physical and chemical properties of HEAs make them attractive candidates for a wide range of applications. The adaptable multicomponent composition of HEAs enables the modulation of surface characteristics and the creation of novel active sites, thereby reducing the activation energy of reactants and intermediates, which significantly enhances their catalytic efficiency [117, 118]. Additionally, the high entropy and slow diffusion characteristics of HEAs contribute to their long-term stability, making them promising materials for energy storage and corrosion-resistant coatings. Over the past few decades, the application of HEAs has expanded significantly across various fields. This section will focus on the latest advancements in their applications, including electromagnetic absorption, energy storage, gas sensors, biomedical applications, and catalysis.

### Electromagnetic Absorption Material

The continuous evolution and innovation in electronics have led to the widespread adoption of the gigahertz frequency band in numerous electronic devices. This has resulted in a gradual increase in electromagnetic interference, emphasizing the urgent need for the development of materials capable of robust electromagnetic wave absorption within this frequency range. The electromagnetic shielding effect is primarily attributed to two mechanisms: reflection and absorption. To effectively interact with an incident magnetic field, the materials must possess mobile carriers (electrons or holes). Metals and their alloys, which have high electrical conductivity, are frequently employed to enhance the reflective effect in electromagnetic shielding materials. Additionally, effective absorption of electromagnetic wave often requires dense porous structures or cavities that allow the dissipation of incident electromagnetic waves within the material through multiple reflections [119]. Due to their variable elemental composition and excellent electromagnetic properties, HEAs are considered promising materials for electromagnetic shielding applications. The controllable elemental composition and compositional concentration of HEAs permit the adjustment of their dielectric and electromagnetic properties. For instance, the selection of elements such as Fe, Co, and Ni allows for the enhancement of soft magnetic properties and conductivity [120]. As shown in Table 2, HEAs exhibit superior performance compared to other materials when used as electromagnetic wave absorbers. Similarly, the choice of elements such as Cu and Mn optimizes impedance matching and high conductivity, further improving the electromagnetic properties of HEAs.

**Table 2:** Comparison of electromagnetic wave absorption performance of HEAs with other materials

Materials	RL_min_ (dB)	EAB (RL < -10 dB) (GHz)	Thickness (mm)	Loading (wt%)	References
Fe@C	− 64.6	6.27	2.2	15	[119]
Ti_3_C_2_T_x_	− 34.4	4.7	1.5	50	[123]
Ti_3_CNT_x_	− 41.36	4.24	1.5	50	[123]
Fe_3_O_4_	− 25.2	3.5	1.6	–	[120]
FeCoNiCuMn HEAs	− 65.8	7.68	3.0	2	[121]
Mg_0.2_Mn_0.2_Fe_0.2_Co_0.2_Ni_0.2_)Fe_2_O_4_	− 35.1	7.48	2.4	70	[122]
(HfTaVWTiMoNbZrCo)B_2_	− 42.6	7.2	1.5	–	[123]

**Fig. 7:**
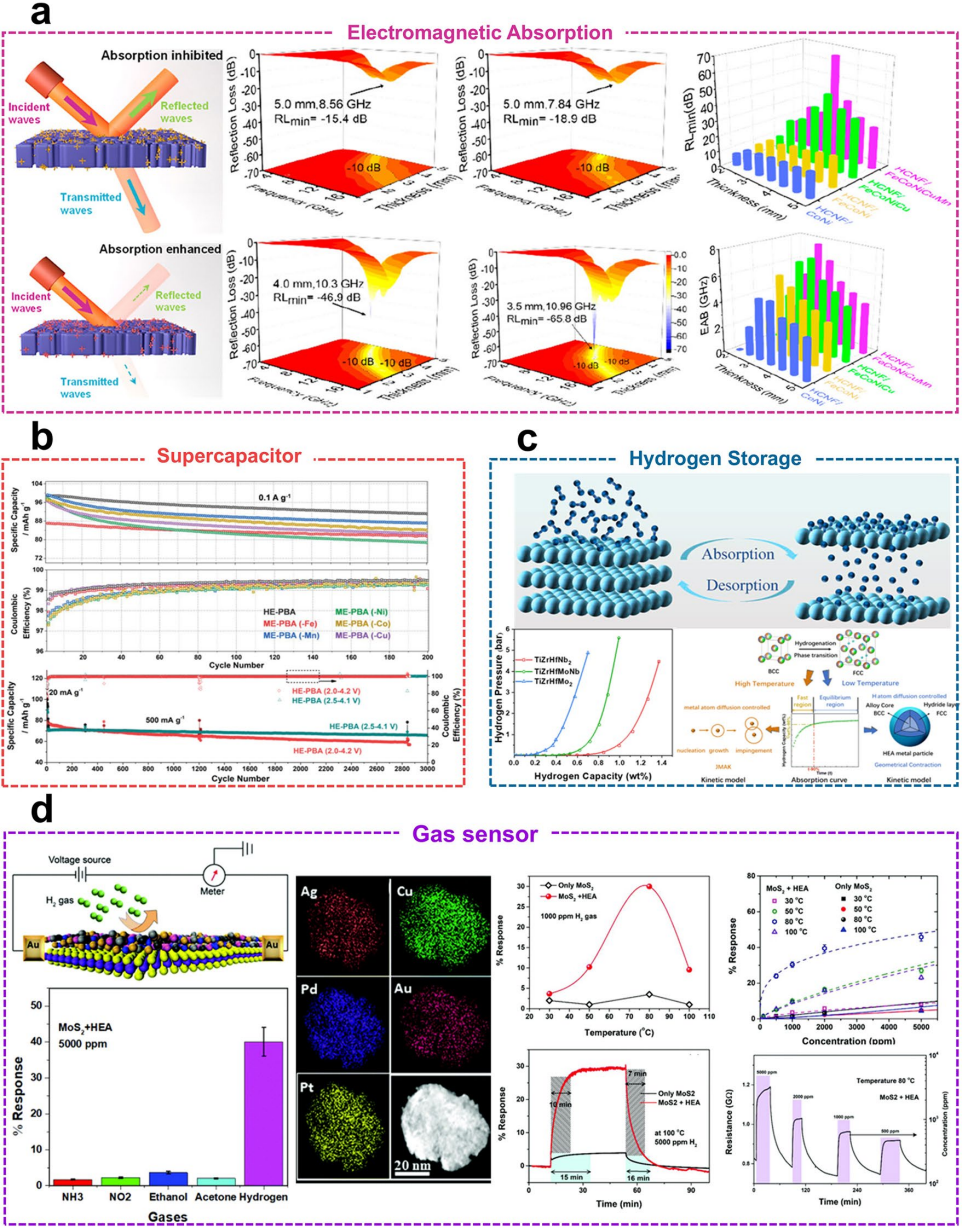
**a** Schematic diagram of electromagnetic wave absorption principle and electromagnetic wave absorption properties of different alloy composites. Reproduced with permission [120, 121]. Copyright 2024, 2022, American Chemical Society, Elsevier. **b** Specific discharge capacity (top) and coulombic efficiency (middle) of different high-entropy Prussian blue electrodes at a current of 0.1 A·g^−1^, and long-term cycling performance at a current of 0.5 A g^−1^ (bottom). Reproduced with permission [34]. Copyright 2021, Wiley. **c** Pressure-composition isotherms at 600 °C and the schematic diagram of hydrogenation phase transition time under high- and low-temperature conditions of TiZrHfMo_x_Nb_2–x_ (x = 0, 1, 2) HEAs powders, respectively. Reproduced with permission [127, 130]. Copyright 2024, 2023, Elsevier, Royal Society of Chemistry. **d** Schematic diagram of H_2_ specific response (left), specific response curves of HEAs-modified MoS_2_ to hydrogen at different temperatures and different concentrations (right). Reproduced with permission [133]. Copyright 2020, Royal Society of Chemistry.

Zhang et al*.* [121] effectively combined electrostatic spinning and Joule heating techniques to integrate FeCoNiCuMn HEAs into honeycomb-like porous carbon nanofibers (HCNF). The combination of HEAs with the HCNF exhibited excellent electromagnetic absorption performance at an ultra-low loading of 2 wt%. This was attributed to the lattice distortion effect of HEAs and the HCNF. The composite material achieved a minimum reflection loss of −65.8 dB and a maximum absorption bandwidth of 7.68 GHz (Fig. 7a). Its dielectric properties improved with increasing entropy, demonstrating its potential as an electromagnetic wave absorber. Currently, ferrite is the most commonly used microwave-absorbing material for combating electromagnetic pollution. However, the limited dielectric loss capability of ferrite presents a major challenge to its large-scale application. To address this issue, Zhou et al*.* [122] fabricated three high-entropy spinel-type ferrite ceramics using the solid-phase synthesis method. The presence of magnetic elements such as Fe, Co, and Ni resulted in excellent magnetic loss properties in all three high-entropy ferrite magnets. Among them, (Mg_0.2_Mn_0.2_Fe_0.2_Co_0.2_Ni_0.2_)Fe_2_O_4_ exhibited the most effective electromagnetic wave absorption, with an optimal effective bandwidth of 7.48 GHz at a thickness of 2.4 mm, ranging from 8.48 to 15.96 GHz. Notably, Gu et al*.* [123] engineered the lattice distortion of high-entropy diboride (HEB) by controlling elemental variables (3–9 elements) and achieved exceptional electromagnetic wave absorption performance, with an effective absorption bandwidth of up to 7.2 GHz at 1.5 mm. The lattice distortion degree of 3–9-element HEB was quantified using a combination of theoretical calculations and XRD, ranging from 0.11 to 0.36 Å. Furthermore, the degree of lattice distortion on electromagnetic wave absorption performance was interpreted from a microscopic perspective, providing valuable insights for the future development of high-performance electromagnetic wave absorbers.

### Energy Storage

HEAs have been widely used in energy storage, particularly multicomponent oxides, which can store more electrical energy than conventional double-layer capacitors through Faradaic redox reactions on their surface. This leads to superior electrochemical activity and at least two orders of magnitude higher electron conductivity compared to single oxides [124, 125]. Consequently, HEAs are regarded as promising candidates for supercapacitors. Brezesinski et al*.* [34] successfully synthesized Na_x_(FeMnNiCuCo)[Fe(CN)_6_] high-entropy Prussian blue analogues by introducing five different metal ions sharing the same nitrogen coordination site within the crystal structure. The quasi-zero-strain mechanism exhibited by this material results in enhanced cycling stability and cycling multiplicity. After more than 3,000 cycles, the material maintained nearly 100% Coulombic efficiency and 68 mAh g^−1^ specific discharge capacity (Fig. 7b). Due to their ultra-fast charging and discharging speeds and high power densities, electrostatic dielectric capacitors have become essential components in advanced electronics. The key challenges for next-generation capacitors include increasing energy density, miniaturization, and integration. In response to these challenges, Lin et al*.* [126] developed a high-entropy dielectric film based on Bi_4_Ti_3_O_12_ by equimolarly introducing Hf and Sn into the Ti sites and La into the Bi sites, forming (Bi_3.25_La_0.75_)(Ti_3-3x_Zr_x_Hf_x_Sn_x_) O_12_ high-entropy dielectric films. This film achieved an energy density of 182 J cm^−3^ and a high efficiency of 78% at an electric field of 6.35 MV cm^−1^. The modulation of atomic configuration entropy contributed to the formation of a stable microstructure in the dielectric films, enhancing breakdown strength, reducing polarization hysteresis, and improving energy storage performance.

Furthermore, HEAs have garnered significant interest in solar vapor power generation and seawater desalination, as they broaden the operating bandwidth and enhance photothermal conversion performance, which is attributed to the increased interband transitions resulting from the fully populated energy regions around their Fermi energy levels [68]. Zhang et al*.* [65] demonstrated the innovative construction of carbon-based core and HEAs shell microstructures, where they confined HEAs in situ within multilayer graphene shells. By leveraging the low thermal conductivity of graphene, they synergized the interband transition of HEAs, significantly enhancing the water evaporation rate (2.66 kg m^–2^ h^–1^) and photothermal conversion efficiency (98.2%). Interestingly, Zhang et al*.* [66] also developed a composite evaporator for seawater desalination, leveraging the unique anisotropic porous structure and hydrophilic surface of balsawood matrices to enable rapid water transport. This synergized with the efficient hydrophobic photothermal performance of HEAs, formed a salt-resistant, high-temperature surface with an absorption rate of  > 97% across the entire solar energy spectrum and an evaporation rate of up to 2.58 kg m^–2^ h^–1^. Moreover, such composited evaporator demonstrated efficient long-term recyclability in high-salinity water. When evaluated over a long cycle of desalination in saline water with salinity up to 20 wt%, the composite evaporator maintained stable solar desalination performance for up to 10 cycles, with an average evaporation rate remaining as high as 1.65 kg m^–2^ h^–1^. This work introduces entirely new application scenarios for HEAs in the development of high-performance photothermal energy converters and desalination devices, significantly expanding the potential of HEAs for practical applications.

In addition to the applications mentioned above, HEAs have garnered increasing attention as potential hydrogen storage materials. Multi-element alloys with varying atomic sizes and high concentrations inevitably cause localized deformation, inducing lattice distortion effects that enhance hydrogen storage. Wu et al*.* [127] modulated the lattice distortion in TiZrFeMnCrV_x_ (*x* = 0.5, 1.0, 1.5, 2.0 at%) HEAs by varying the V content and investigated the impact of lattice distortion on the hydrogen adsorption properties of HEAs. The lattice distortion in HEAs gradually increases with rising V content, leading to enhanced hydrogen storage properties. The highest hydrogen storage performance is observed at *x* = 1.0%, with the hydrogen absorption capacity increasing to 1.91 wt%. The best dehydrogenation performance is achieved with hydrogen release beginning at 151.33 °C and ending at 346.36 °C. This research demonstrates excellent hydrogen storage performance by leveraging the lattice distortion effect in HEAs, paving the way for further advancements in HEA applications. The number of hydrogen atoms that can be stored in metal hydrides is typically lower than the number of available interstitial sites, limiting their hydrogen storage capacity [128]. Therefore, enhancing the occupation of hydrogen atoms in interstitial positions is key to improving the hydrogen storage capacity of metallic materials. Vitos et al*.* [129] demonstrated that in FCC HEAs hydrides, hydrogen atoms can occupy octahedral interstices up to 20% (Ti_25_V_25_Nb_25_Ta_25_) and 17.5% (Ti_25_V_25_Nb_25_Zr_25_), respectively. The delocalized electrons of hydrogen atoms in these hydrides lead to anomalous hydrogen occupation, reducing the repulsion between hydrogen atoms and promoting electron localization at interstitial sites. These combined effects significantly increase hydrogen storage density. Moreover, Zu et al*.* [130] investigated the effects of composition and temperature on the hydrogenation properties of TiZrHfMo_x_Nb_2–x_ (*x* = 0, 1, 2) HEAs, adjusting the composition by substituting Mo for Nb. Since Mo has a lower hydrogen affinity than Nb, increasing Mo concentration enhances the migration of hydrogen within the lattice. Notably, the hydrogen absorption kinetics of HEAs follow the geometric contraction model at low temperatures, which is controlled by hydrogen atom diffusion, and the nucleation-growth impingement model at higher temperatures, which is controlled by metal atom diffusion (Fig. 7c).

### Gas Sensor

The importance of gas sensing technology continues to grow in fields such as environmental monitoring and industrial production. HEAs with diverse composition are capable of generating numerous active sites that interact with gas, exhibiting exceptional capabilities in gas adsorption and charge transfer. This indicates the immense potential of HEAs as highly sensitive and selective gas sensors. For instance, the selective detection of CH_4_ gas in industrial production is crucial for preventing catastrophic events. Singh et al*.* [131] developed a single-phase high-entropy oxide chemoresistor for highly selective CH_4_ detection. This sensor operates at a power consumption of only 50 nW, which is significantly lower than that of other commercially available CH_4_ sensors. Additionally, it demonstrates ultra-low detection limits with a sensitivity of 25 ppm. Furthermore, the sensor offers several advantageous properties, such as high sensitivity, rapid response and recovery times, and long-term stability, making it ideal for real-time monitoring of CH_4_ levels in the atmosphere. In another advancement, Biswas et al*.* [132] developed an Al_70_Co_10_Fe_5_Ni_10_Cu_5_ HEAs nanosheet with an ultrathin edge thickness of approximately 6 nm. This nanosheet exhibits anisotropic electron transport properties and ultra-sensitive detection of NO_2_ gas at 100 °C, with a detection limit ranging from 1 to 100 ppm and a gas response of 46%. Additionally, Kamble et al*.* [133] designed a AuAgCuPdPt HEAs-modified MoS_2_ nanosheet that enhances hydrogen adsorption response at 80 °C. This enhancement was achieved by combining metal elements with strong hydrogen absorption capabilities (Pd and Pt) and weak hydrogen absorption properties (Au and Ag), forming nano-junctions on MoS_2_ sheets (Fig. 7d).

### Biomedical Materials

Entropy-mediated design strategies for polymetallic nanozymes have the potential to significantly reduce the activation energy of reactions, representing an innovative approach to enhancing the efficiency of heterogeneous catalysis. As a result, conformational entropy-driven HEAs are emerging as a promising solution to the catalytic efficiency bottleneck in nanozymes. Moreover, the synergistic effects of multifunctional active sites significantly enhance their applicability in biomedical fields. For example, Liang et al*.* [20] successfully developed a POD-like PtPdRuRhIr HEAs capable of catalyzing the decomposition of endogenous H₂O₂ into highly toxic **·**OH radicals, while synergizing with photothermal effects, resulting in a highly efficient cancer cell-killing rate against breast cancer cells. In vitro cellular experiments confirmed the good biocompatibility of HEAs. When the HEAs concentration was increased to 100 µg mL^−1^, a significant cancer cell-killing effect was observed, reducing the survival rate of 4T1 and Hep G2 cells to approximately 75%. At the same concentration, normal endothelial cells (IEC-6) exhibited over 90% survival, indicating lower cytotoxicity. While exhibiting a high cancer cell-killing rate, the metabolic residues of various heavy metal elements in HEAs in organs such as the heart, liver, spleen, lungs, and kidneys were found to be nearly zero, significantly lower than the accumulation in the tumor, demonstrating highly efficient tumor targeting. Furthermore, no obvious damage was observed in the tissue sections, further confirming the high biocompatibility of HEAs. This study provides new opportunities for further investigation into the potential of HEAs as catalysts in biomedical applications. Ascorbic acid (vitamin C, VC) is essential in mitigating cellular damage caused by an imbalance between reactive oxygen species and antioxidants. However, both deficiency and excess levels of VC can negatively affect the body. Therefore, Khakbiz et al*.* [134] innovatively prepared LDH NPs, LDH@SiO_2_, and ZIF-8 NPs-encapsulated NiCoCrFeMn HEAs carriers by electrophoretic deposition, and embedded VC between the encapsulated layers via ion exchange to achieve the precise controlled release of VC. Compared to the other coatings, the LDH@SiO_2_-coating released over 5% more VC and demonstrated an appropriate biological response in MG-63 cells. This research demonstrates that Zn/Al-LDH@SiO_2_-coated layers are promising candidates for delivering VC to the body and facilitating favorable interactions between bone tissue and HEA implants. This research offers valuable insights into novel methods for surface functionalization and coating of HEAs, significantly expanding their potential for biological applications.

**Fig. 8:**
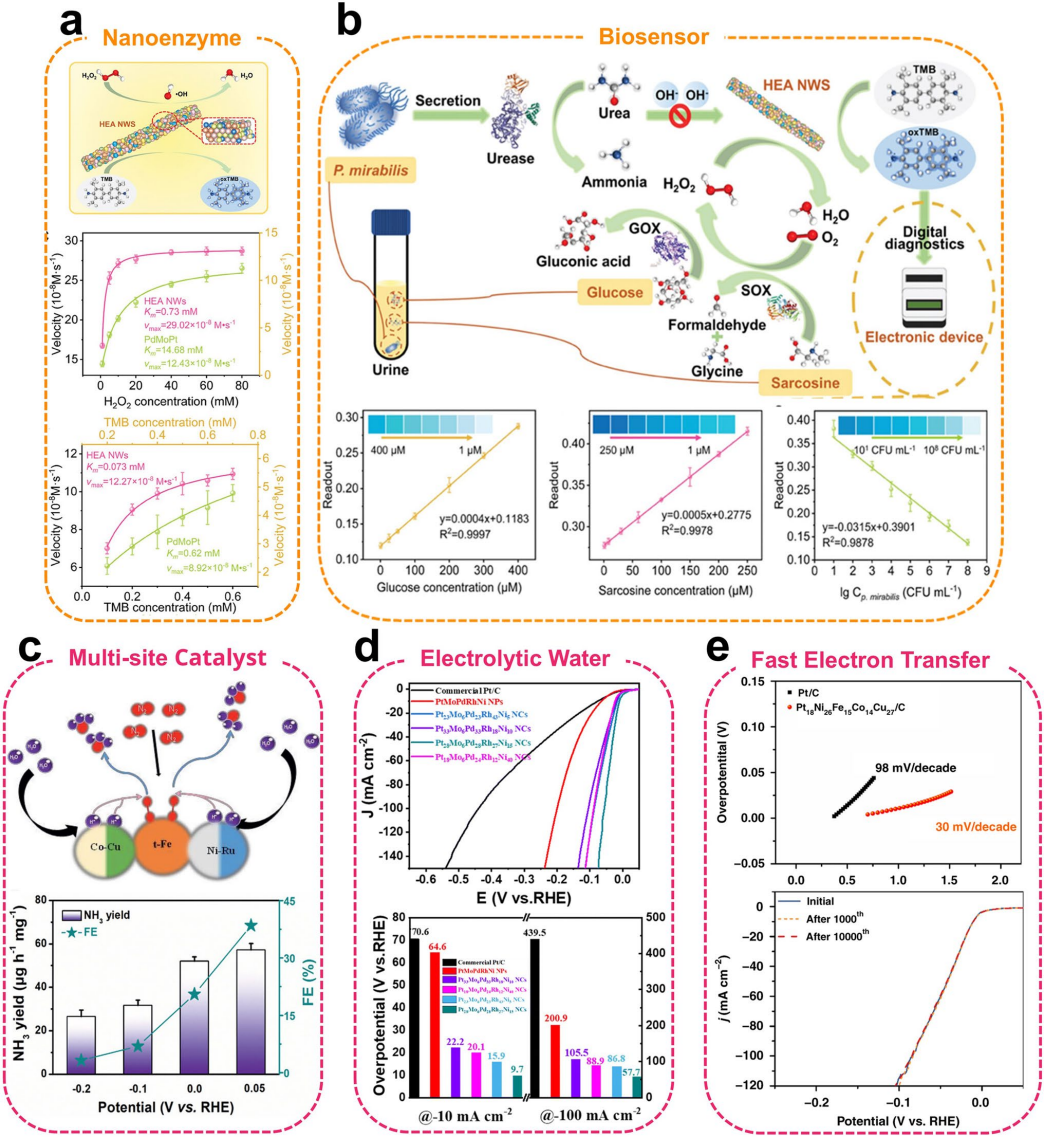
**a** Schematic representation of the POD-like catalytic process of HEAs nanozymes (top), Michaelis–Menten curves of HEAs and PdMoPt ternary alloys for different concentrations of H_2_O_2_ (middle) and TMB (bottom), respectively. **b** Digital colorimetric detector developed based on HEAs nanoenzymes for the detection of glucose, sarcosine, and *P. mirabilis* in urine. Reproduced with permission [137]. Copyright 2024, Wiley. **c** Schematic representation of possible multisite electrocatalytic mechanisms illustrating the enhancement of NRR activity by RuFeCoNiCu HEAs at low overpotentials (top), along with NH_3_ yields and Faradaic efficiency at each specified potential in 0.1 M KOH (bottom). Reproduced with permission [140]. Copyright 2021, Wiley. **d** HER polarization curves in 1.0 M KOH solution (up) and overpotentials at current densities of -10, -100 mA cm^−2^ (down) of HEAs nanocrystals, HEAs nanoparticles and commercial Pt/C, respectively. Reproduced with permission [146]. Copyright 2023, Royal Society of Chemistry. **e** Tafel slopes (top) and HER polarization curves for different CV cycles of HEAs (bottom). Reproduced with permission [147]. Copyright 2020, Springer Nature.

The results demonstrate that Pt effectively lowers the reduction temperature of other elements, thereby facilitating the simultaneous reduction of multiple elements under low-temperature conditions [135, 136]. Consequently, Tang et al*.* [137] successfully prepared a novel PdMoPtCoNi HEAs nanoenzyme with excellent substrate affinity and a high catalytic rate (Fig. 8a). The strong *d*-orbital coupling of the constituent elements in HEAs modifies the electron distribution near the Fermi energy level (*E*_F_). Furthermore, the *d*-electrons of Co and Ni significantly increase the electron density near the *E*_F_, thus optimizing the *OH dissociation energy on PdMoPt and enhancing catalytic performance. Moreover, Tang et al*.* employed the synthesized nanozymes as biosensors to successfully detect glucose, sarcosine, and *P. mirabilis* in urine samples (Fig. 8b). In response to bacterial infections, Shen et al*.* [138] successfully developed high-entropy MXenes (HE MXenes) with strong oxidase-like activity (K_m_ = 0.227 mM) and entropy-mediated photothermal efficiency (65.8%) in the NIR-II region. HE MXenes exhibit excellent biocatalytic properties and can be used as nanotherapeutic agents for the effective treatment of bacterial keratitis and subcutaneous abscesses caused by methicillin-resistant *Staphylococcus aureus*, due to NIR-II-enhanced intrinsic oxidase-like activity.

### Catalyst Materials

Catalysts play a crucial role in industrial production, significantly reducing energy consumption, enhancing reaction selectivity and rates, and altering radical reactions. The elemental diversity and structural advantages of HEAs offer a wide compositional space to modulate surface electronic structures and catalytic properties, thereby demonstrating significant potential for catalytic applications. Thus, the electrocatalytic reaction of HEAs is a multistep process involving product cascades, interactions, mediation, and electron transfer. The unique interactions among their numerous neighboring elements form the basis of the multisite active centers [139]. For example, the RuFeCoNiCu HEAs synthesized by Wang et al*.* [140] for the first time at low temperatures (≤ 250 °C) under atmospheric-pressure exhibited an NH_3_ yield of 57.1 µg h^–1^ mg_cat_^−1^ (0.05 V) in 0.1 M KOH (The bottom of Fig. 8c), along with excellent nitrogen reduction reaction (NRR) activity in 0.1 M Li_2_SO_4_ (52.6 µg h^–1^ mg_cat_^−1^), Na_2_SO_4_ (47.2 µg h^–1^ mg_cat_^−1^), and HCl (37.1 µg h^–1^ mg_cat_^−1^). Notably, its catalytic activity remained stable for up to 100 h of testing, demonstrating excellent chemical stability. DFT calculations further indicate that Fe in the alloy serves as the optimal site for N_2_ adsorption and activation, while Co–Cu and Ni-Ru couples exhibit efficient hydrogenation at lower overpotentials. HEAs exhibit outstanding multisite NRR activity across a wide pH range, revealing a novel mechanism in NRR research (The top of Fig. 8c). Catalysis involves interactions between reacting molecules and the catalyst surface, including charge transfer, adsorption, decomposition, and molecular transformation. Consequently, the catalytic performance primarily depends on the surface properties, including its chemical, geometric, and electronic structures [141, 142]. To emphasize the significant contribution of HEAs to the enhancement of catalytic performance, a selection of prominent catalytic reactions has been presented in this section, along with an illustration of other potential applications of HEAs in catalysis.

**Fig. 9:**
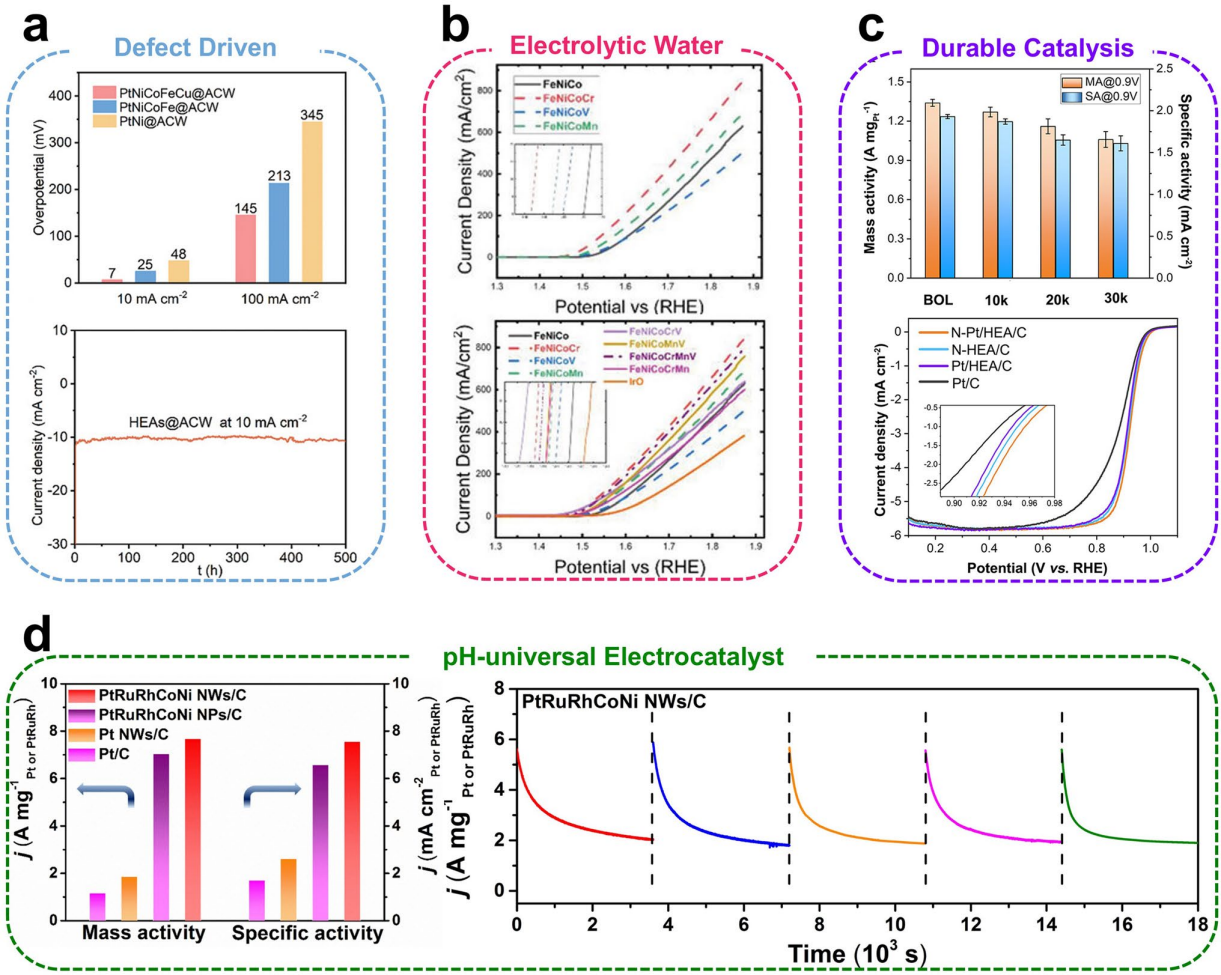
**a** Overpotentials of different catalysts at 10 and 100 mA cm^−2^ (top), chronoamperometry test of PtNiCoFeCu HEAs in KOH electrolytes at 10 mA cm^−2^ (bottom). Reproduced with permission [149]. Copyright 2024, Wiley. **b** LSV scans comparing the impact of Cr, V, and Mn incorporation on the catalytic performance of quaternary high-entropy oxides (top), and LSV scans comparing the difference in catalytic performance of sequentially alloyed quaternary, quinary, and senary high-entropy oxides with state-of-the-art IrO_2_ catalysts (bottom). Reproduced with permission [152]. Copyright 2022, Wiley. **c** Specific activity and mass activity of the N-doped PtCoFeNiCu HEAs catalyst at BOL and different potential cycles (top), ORR polarization curves of different catalysts in an O_2_-saturated 0.1 mol L^−1^ HClO_4_ (bottom). Reproduced with permission [156]. Copyright 2024, American Chemical Society. **d** Mass activity and specific activity for EOR (left), long-time durability of PtRuRhCoNi HEAs (right). Reproduced with permission [160]. Copyright 2022, Elsevier.

*Electrolytic water* Hydrogen is widely recognized as one of the cleanest and most sustainable energy sources, and hydrolysis is considered one of the most efficient and environmentally friendly technologies for hydrogen production under mild conditions [143]. The compositional and structural diversity of HEAs allows for broad modulation of surface electron properties, enabling excellent HER performance under both acidic and alkaline conditions. Therefore, the relationship between constitutive composition and catalytic activity is closely intertwined. A widely adopted strategy involves selecting highly active metals and pairing them with other elements based on their electronic structures to effectively harness the cocktail effect of HEAs [144, 145]. Wang et al*.* [146] were the first to report PtMoPdRhNi HEAs with robust d-d electronic interactions. Additionally, Pt_28_Mo_6_Pd_28_Rh_27_Ni_15_ demonstrated the highest alkaline HER activity by strategically adjusting atomic occupancy for each element, achieving an ultra-low overpotential of 9.7 mV at a current density of -10 mA cm^−2^. Besides the effects of morphology, the complex composition of HEAs results in differential contributions from their elements to catalysis, promoting electron transfer between sites and stabilizing catalytic intermediates (Fig. 8d). Wang et al*.* [147] synthesized small-sized (3.4 nm) and homogeneous Pt_18_Ni_26_Fe_15_Co_14_Cu_27_ HEAs that exhibited a low overpotential of 11 mV at 10 MA cm^−2^, demonstrating exceptional cycling stability after 10,000 CV tests (Fig. 8e). During the catalytic process, Pt atoms serve as electron reservoirs for HER, facilitating electron transfer, while Ni and Co atoms primarily influence electronic activity near the Fermi energy level. Corrosion of metal-based catalysts in various electrolytes poses a significant challenge to developing highly efficient HER catalysts. Notably, the successful synthesis of Pt_18_Ni_26_Fe_15_Co_14_Cu_27_ HEAs not only demonstrates a multi-active site and fast site-to-site electron-transfer mechanism but also represents the first reported synthesis of oil-phase colloidal HEAs under mild conditions. This breakthrough introduces a novel methodology and a new research direction for colloidal alloy materials, laying the foundation for the facile synthesis and multi-field applications of other HEAs. To address this, Bi et al*.* [148] developed Ni_20_Fe_20_Mo_10_Co_35_Cr_15_ HEAs that exhibit high corrosion resistance, activity, and stability in both acidic and alkaline electrolytes. This catalyst achieved an overpotential of 107 mV in acidic electrolyte and 172 mV in alkaline solution at 10 mA cm^−2^, representing an improvement over biphasic catalysts. It is worth noting that Gan et al*.* [149] reported a defect-driven surface engineering approach to fabricate highly active and durable PtNiCoFeCu HEA electrocatalysts via rapid quenching treatment. Their results demonstrated excellent cycling stability, with an overpotential of 7 mV at 10 mA cm^−2^ (The top of Fig. 9a) and a cycle life of 500 h (The bottom of Fig. 9a). The high entropy and slow diffusion effects of HEAs promote the formation of a single solid solution, preventing potential differences between multiphases. This not only enhances the corrosion resistance of HEAs but also avoids phase separation during the electrocatalytic process. To assess the stability of HEAs as catalysts, quaternary FeCoNiCu and quintuple PtFeCoNiCu HEAs were designed [150]. The PtFeCoNiCu alloy demonstrated improved stability due to multi-elemental modulation and entropy stabilization effects, exhibiting negligible performance degradation in the 100 h HER stability test. This enhanced stability can be attributed to the homogeneous dispersion of strong metallic bonds and effective modulation of Pt’s electronic structure.

The OER is another crucial half-reaction in water electrolysis. Metallic materials containing Ir and Ru are considered the most effective catalysts for the OER. Compared to the two-electron reaction of HER, OER involves a four-electron transfer, making its high kinetic overpotential a limiting factor in the reaction rate. Key factors in designing OER catalysts include enhancing electron transport efficiency at the catalytic center and improving the absorption efficiency of active intermediates during the OER process. Huang et al*.* [151] successfully synthesized FeCoNiRu HEAs, where intrinsic hollow active sites significantly improved OER performance. Moreover, high catalytic activity was sustained after a 40h stability test. Additionally, the significant electronegativity difference between Ru and the other constituent elements generates numerous active sites on the surface of HEAs, enabling them to function as both cathode and anode during electrolysis. Notably, Li et al*.* [152] investigated the contributions of various constituent elements in HEAs to OER performance. Their findings revealed that adding Cr promotes higher oxidation states of active elements (Co, Ni, Fe) and maximizes the adsorption of highly oxidizing species, thus enhancing OER activity (Fig. 9b). Specifically, compared to single- or bimetallic catalysts, HEAs offer significant advantages in terms of elemental combinations. The incorporation of abundant and widely available elements, such as Fe, Co, Ni, and Cu, not only enhances catalytic activity across a broad pH range but also creates multisite catalytic centers and a rich electronic structure. Furthermore, this approach reduces the reliance on precious metals like Pd, Pt, Ru, Rh, and Ir, thereby lowering costs and making HEAs more promising for practical applications.

**Fig. 10:**
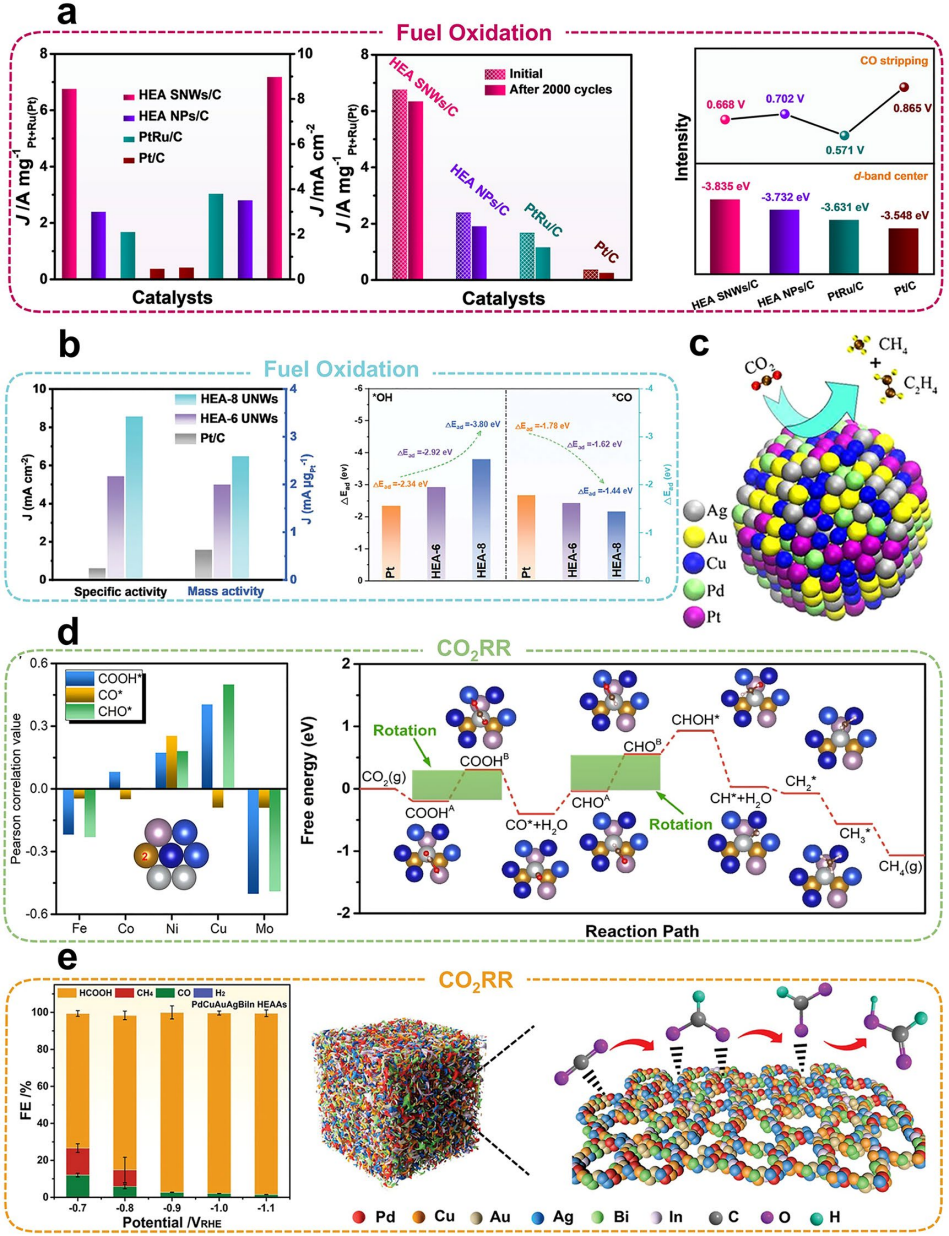
**a** Comparison of normalized mass activity and specific activity at 50 mV vs. RHE overpotential (left), before and after 2000 cycles of ADTs (middle). Comparison of d-band center positions and CO stripping potential of different catalysts (right). Reproduced with permission [154]. Copyright 2021, Springer Nature. **b** Comparison of catalytic performance between HEAs and commercial Pt/C (left), adsorption energy of *OH and *CO on different catalysts (right). Reproduced with permission [160]. Copyright 2024, Wiley. **c** Schematic diagram of HEAs as CO2RR catalysts. Reproduced with permission [167]. Copyright 2020, American Chemical Society. **d** Pearson correlation values for the adsorption of COOH*, CO*, and CHO* by different atoms of HEAs (left), CO_2_RR reaction process on AS1 (right). Reproduced with permission [168]. Copyright 2022, American Chemical Society. **e** Reduction potential-dependent Faraday efficiency measurements of HEAAs (left), schematic representation of HEAAs promoting the generation of HCOOH products (right). Reproduced with permission [169]. Copyright 2023, Wiley.

*Fuel oxidation* The conversion of chemical energy from fuels to electricity is a promising energy conversion technology that can effectively reduce dependence on fossil fuels and mitigate irreversible environmental damage. In fuel cells, output power and energy conversion efficiency depend on the fuel oxidation reaction at the anode [153]. Thus, developing suitable materials for anodes can reduce activation energy and achieve satisfactory energy conversion efficiency. When five or more elements are homogeneously mixed, the system gains a higher entropy of mixing, leading to the formation of a uniform solid solution and enhanced thermodynamic stability of the catalytic system. Additionally, diffusion and phase transitions are influenced by the varying potential energies and diffusion rates of different elements. This disparity hinders the diffusion process, helping to preserve the integrity of the HEAs crystal structure. As a result, HEAs can maintain high catalytic activity while retaining structural stability, thereby extending their recyclability. The high-energy density and pollution-free features of hydrogen fuel cells make the promotion of the hydrogen oxidation reaction (HOR) crucial for increasing fuel cell energy density. PtRu-based metal alloys are widely used in HOR, and adding other elements to form HEAs produces catalysts with high catalytic activity and excellent corrosion resistance. Consequently, the development of HEAs with excellent electrocatalytic performance has attracted significant interest. Huang et al*.* [154] reported PtRuNiCoFeMo HEAs for alkaline HOR with a mass specific activity of 6.75 A mg_Pt+Ru_^−1^ and 8.96 mA cm^−2^. Results from 2,000 cycle durability tests showed no significant performance degradation. Compared to commercial PtRu/C and Pt/C catalysts, HEAs exhibit mass activities that are 4.1 and 19.8 times higher, respectively, and demonstrate excellent resistance to CO poisoning (Fig. 10a). Furthermore, Luo et al*.* [155] reported the development of PdNiRuIrRh HEAs catalysts with a mass activity of 3.25 mA μg^−1^ in alkaline HOR, representing an eightfold improvement over commercial Pt/C catalysts. Notably, Sasaki et al. [156] reported a nitrogen-doped PtCoFeNiCu HEA electrocatalyst that retained 79.1% of its mass activity and maintained significant ORR activity after 30,000 cycles (The top of Fig. 9c). It exhibited a high mass activity of 1.34 A mg_Pt_^−1^ and a specific activity of 1.93 mA cm^−2^ at 0.9 V, which were 7.4 and 6.2 times higher, respectively, than those of commercial Pt/C catalysts (The bottom of Fig. 9c). This remarkable enhancement in catalytic activity may be attributed to the synergistic effects of high entropy, multiple M–N bonds, and Pt-rich shells. Specifically, the mass activity of the Cu- and Fe- single-atom catalysts was only twice as high in alkaline conditions and five times higher in acidic conditions compared to commercial Pt/C, with a cycling stability of just 300 h [157]. The catalytic activity remained significantly lower than that of HEAs composed of the same elements, despite their exceptionally large specific surface area of 2883.7 m^2^ g^−1^. This discrepancy is likely due to the higher concentration of active atoms and the greater electron-transfer efficiency of HEAs under identical conditions compared to single atomic catalysts. The long-term cyclic stability of HEAs can be attributed to differences in the diffusion rates of their constituent elements. The diffusion behavior of these alloys results from the coordinated interaction of multiple atomic species, where some elements exhibit lower activity and are less likely to migrate into vacancies. Consequently, the slow-diffusing elements act as a limiting factor for overall diffusion within the alloy. This slow diffusion effect enhances the phase stability, high-temperature strength, and creep resistance of HEAs, ultimately contributing to their exceptional durability and structural stability throughout the electrocatalytic process.

Liquid fuels are easier to store and transport than hydrogen, and thus high-energy–density liquid fuels are used in place of hydrogen to drive anode oxidation reactions. The methanol oxidation reaction (MOR) and ethanol oxidation reaction (EOR) are fundamental anode reactions in methanol fuel cells, but they exhibit low reaction kinetics. HEAs can alter the electronic properties of catalysts and strengthen the binding of intermediates, resulting in remarkable electrocatalytic performance [158, 159]. Recently, Wang et al*.* [160] developed an ultrasmall PtRuRhCoNi HEAs (1.6 nm) that functions as a pH-universal electrocatalyst by leveraging the complementary effects of its electronic structure. It exhibits an EOR activity of 7.68 A mg_PtRuRh_^−1^ and a high selectivity of 78% for the C_1_ product (The left of Fig. 9d), which was the highest selectivity ever reported for an EOR catalyst. Additionally, its MOR activity reaches 6.65 A·mg_PtRuRh_^−1^, making it one of the most effective MOR catalysts reported. Notably, the catalyst demonstrated negligible activity decay and CO toxicity resistance even after 2,000 cycles. Notably, the catalytic activity of HEAs can be reversibly restored by replacing the electrolyte with a working solution in cyclic CA tests, without significant attenuation in performance (The right of Fig. 9d). This unique elemental composition endows HEAs with a strong electronic complementary effect, leading to exceptional catalytic activity across a wide pH range. These findings provide valuable insights into the development of multi-point electrocatalysts for broad pH-universal. Qin et al*.* [161] successfully synthesized PtRuNiCoFeGaPbW ultrathin HEA nanowires that induce *p*-region metals (Ga and Pb) with distinctive *p*-*d* orbital hybridization. The specific and mass activities of HEAs for MOR were enhanced by 15.0-fold and 4.2-fold, respectively, compared to commercial Pt/C. Notably, the MOR process follows a completely “non-CO” pathway, effectively preventing the generation and adsorption of intermediate CO (Fig. 10b). Meanwhile, Huang et al*.* [59] developed PdPtCuPbBi ultrathin HEA nanorings, which demonstrated 25.6-fold and 16.3-fold increases in mass activity compared to commercial Pd/C and Pt/C catalysts in EOR, respectively.

Compared to other fuels, formic acid's low toxicity, ease of storage, and transportation have enabled its extensive use in fuel cells. Huang et al*.* [162] successfully constructed PtBiPbNiCo high-entropy hexagonal nanoplates featuring a unique PtBiPd medium-entropic core and PtBiNiCo high-entropy atomic shell structure, which exhibited a specific activity of 27.2 mA cm^−2^ and a mass activity of 7.1 A mg_Pt_^−1^. Additionally, the specific and mass activities of HEAs were shown to be 133-fold and 56-fold higher than those of commercial Pt/C catalysts, respectively. Notably, the catalyst exhibits the highest performance to date, making it the most efficient Pt/Pd-based methanol oxidation catalyst.

*Carbon dioxide reduction reaction (CO*_*2*_*RR)* Electrocatalytic CO_2_RR has the potential to produce high-value chemicals such as C_1_ (CO, CH_4_, HCOOH, HCHO, CH_3_OH), C_2_ (C_2_H_2_OH, CH_3_COOH, C_2_H_4_), and C_3+_ products. This process can help alleviate the energy crisis and mitigate the greenhouse effect caused by CO_2_ [163]. However, the high thermodynamic stability of CO₂ leads to multiple proton-electron coupling transfer steps during the reduction process. As a result, the production selectivity and Faraday efficiency of x ≥ 2 products are low, and the lack of catalyst activity for the C–C coupling reaction presents a major challenge to forming x ≥ 2 products where x represents the number of carbon atoms in reduction products [164–166]. Thus, designing highly selective and efficient electrocatalysts for CO_2_RR to produce high-value-added C_2+_ products offers significant economic advantages. Therefore, when designing high-entropy electrocatalysts, selecting as many elements as possible with high catalytic activity for the same product can selectively enhance the yield of high-value-added products. Specific transition elements have been reported to exhibit effective catalytic activity in the CO_2_RR process (Fig. 10c). For instance, Cu-based materials exhibit strong binding with *CO and reduce CO_2_ to C_2+_ products, achieving a balance between CO_2_ activation and the hydrogenation barrier of *CO. Additionally, Cu has a low affinity for H, which inhibits the competitive generation of H_2_ and enhances catalyst selectivity [167].

Rossmeisl et al*.* [168] presented a probabilistic and unbiased method for accurately predicting the adsorption energies of CO and H at all sites on the (111) crystalline surfaces of CoCuGaNiZn and AgAuCuPdPt HEAs. This method increases the frequency of desired sites by enhancing the probability of the corresponding locations. For instance, enhancing the probability of the weaker site for hydrogen adsorption inhibits the formation of hydrogen molecules. Similarly, increasing the probability of the stronger site for CO adsorption facilitates CO reduction, thereby selectively enhancing the yield of the desired products. Similarly, Hu et al*.* [169] synthesized Au single atomic carbon nanocages using a mild dip-drying method. Due to the highly coordinated structure of noble metal single atomic, the Au single atomic catalysts exhibited excellent activity of 3319 A g_Au_^−1^ at −1.0 V. Additionally, the H_2_/CO ratio could be precisely controlled by adjusting the external voltage within the range of 0.4–2.2. The Au single atomic active centers were coordinated with N atoms, and the catalytic activity was significantly enhanced by the smooth transition between secondary and quaternary coordination states. Notably, a substrate-mediated single-atom formation strategy is employed to fabricate high-entropy single atomic alloys with a transition metal single-atom loading density of up to 49 wt%. This process leverages the reversible redox reaction between the ionic interfaces of MoS_2_/MoSe_2_ and the transition metal, generating abundant structural vacancies on the MoS_2_/MoSe_2_ surface. These vacancies serve as coordination sites for transition metal single atoms, facilitating the formation of high-density high-entropy single atomic. Excitingly, the high-entropy single atom (PtRuRhPdRe-MoSe_2_) exhibits an overpotential of only 35 mV at a current density of 10 mA cm^−2^, surpassing most reported advanced single atomic catalytic systems. Its steady-state polarization curves reveal that cell voltages of only 1.82 and 1.98 V are required to achieve current densities of 1 and 1.5 A cm^−2^, respectively. Furthermore, it demonstrates exceptional stability, with negligible voltage decay (only 10 mV) over nearly 400 h of water electrolysis. These results clearly validate the outstanding catalytic activity and stability of high-entropy single atoms [170]. Additionally, Chen et al*.* [171] designed FeCoNiCuMo HEAs for simulations to predict the adsorption energies of COOH*, CO*, and CHO*. The rotation design of COOH* and CHO* on the surface of HEAs resulted in excellent catalytic performance, with a limiting potential of 0.29–0.51 V (Fig. 10d). High-entropy alloy aerogels (HEAAs) represent a promising novel catalyst for CO_2_RR, combining the advantages of both HEAs and aerogels. Liu et al*.* [172] developed PdCuAuAgBiIn HEAAs that achieved a Faraday efficiency of nearly 100% for C_1_ products in the voltage range of −0.7 to −1.1 V using a freeze-thawing method. The maximum Faraday efficiency reached 98.1% for formic acid at −1.1 V versus the reversible hydrogen electrode, surpassing the efficiency of both HEAs and aerogel catalysts used independently (Fig. 10e). The electronic structure of various metals can be modulated through strong interactions between unsaturated sites, which also facilitate the adsorption and desorption of HCOO* intermediates on the catalyst surface. This process enhances the selective generation of HCOOH.

## Conclusions and Outlook

Since the concept of HEAs was initially proposed in 2004, they have demonstrated remarkable compositional and structural flexibility, inherent reactivity, and excellent stability, presenting significant potential for applications across multi-fields. This review begins by introducing the fundamental concepts associated with HEAs. It then summarizes the latest research developments in the synthesis of nanoscale HEAs, focusing on several commonly employed strategies. Additionally, it emphasizes the significant challenges encountered in the synthesis of nanoscale HEAs, including homogeneous mixing of multiple elements, migration and aggregation of elements at high temperatures, and the simultaneous reduction of constituent elements. Finally, this review presents a comprehensive analysis of the advanced advantages of HEAs for multi-field applications, emphasizing significant application trends associated with nanosizing and multidimensionalization. Despite significant advancements in nanoscale synthesis strategies for HEAs, their multi-field applications remain in nascent stages, and prospective large-scale applications face enormous challenges. In the light of the aforementioned considerations, this review offers the following outlook:

1. The controllable fabrication and on-demand personalization of HEAs: Despite numerous reports on the controllable preparation of HEAs, achieving a controllable morphology remains limited due to the inherent complexity of their composition and structure. Conventional strategies for structural modulation of materials, including defect engineering, surface modification, and interfacial engineering, have yet to be fully applied to HEAs. Notably, achieving surface manipulation of nanoscale HEAs is particularly challenging due to the potential differences and electronegativity discrepancies among the constituent elements, further complicating the process of fine structural modulation. Therefore, advancements in controllable and green preparation methods and on-demand personalization will create opportunities for low-cost production and large-scale applications of HEAs.
2. The thorough investigation of the mechanisms underlying the growth of HEAs: The complex elemental composition of HEAs leads to unique growth mechanisms. Therefore, a comprehensive understanding of the relationship between elemental composition and growth mechanisms is crucial for developing high-performance HEAs. This necessitates the urgent development of advanced microcharacterization techniques. Furthermore, in situ characterization techniques are commonly utilized to investigate the structural evolution and growth processes of materials, providing valuable insights into the growth mechanisms and surface-active sites of HEAs.
3. High-throughput computational and theoretical prediction techniques: The extensive range of metallic elements results in limitless possible combinations. Consequently, screening for high-performance target HEAs is often conducted using randomized experimental methods. The application of high-throughput computational and theoretical prediction techniques enables accelerated screening and data mining, thus facilitating a more efficient exploration of multi-element composition HEAs.
4. The expansion of application for HEAs: The current status of multi-field applications of HEAs remains in its infancy. Although satisfactory performance has been observed in electrocatalysis and electromagnetic shielding, significant barriers remain to be overcome before HEAs can be deemed economically viable. Consequently, the primary challenges that must be addressed include the development of cost-effective high-performance HEAs and the establishment of large-scale preparation methods.
